# Encapsulation of Polyphenolic Preparation in Gelatin Fruit Jellies Slows the Digestive Release of Cholinesterase Inhibitors In Vitro

**DOI:** 10.3390/antiox14050535

**Published:** 2025-04-29

**Authors:** Dominik Szwajgier, Ewa Baranowska-Wójcik, Wirginia Kukula-Koch, Katarzyna Krzos

**Affiliations:** 1 Department of Biotechnology, Microbiology and Human Nutrition, University of Life Sciences in Lublin, Skromna 8, 20-704 Lublin, Poland; ewa.baranowska@up.lublin.pl; 2 Department of Pharmacognosy with Medicinal Plants Garden, Medical University of Lublin, 1 Chodzki Str., 20-093 Lublin, Poland; wirginia.kukula-koch@umlub.pl; 3 Invent Farm Sp. z o.o., Ul. Karpacka 66, 20-868 Lublin, Poland

**Keywords:** anticholinesterase activity, anti-inflammatory activity, antioxidant activity, neurodegeneration, encapsulation, Rosaceae fruits, Ericaceae fruits

## Abstract

Peach, apricot, chokeberry, blueberry, cranberry, raspberry, and wild strawberry fruits were used to create a polyphenolic preparation (PP) after enzyme-assisted extraction, ultrafiltration, and concentration. The composition of PP was determined using LC-MS. Gelatin jellies produced with PP, as well as liquid PP, were “digested” in an in vitro model. The entrapment of PP in the gelatin matrix delayed the release of total polyphenolics, flavonoids, flavanols, condensed tannins, and anthocyanins (predominantly during the “small intestinal” phase). PP entrapped in the jelly more effectively (*p* < 0.05) decreased the activity of acetylcholinesterase, butyrylcholinesterase, cyclooxygenase-2 and catalase (during the “small intestinal” phase). However, no significant (*p* < 0.05) effects on superoxide dismutase, glutathione peroxidase, and glutathione reductase activities were observed. FRAP, CUPRAC, HORAC, oxidation of linoleic acid, and ABTS-reducing activities were higher during the “intestinal” phase; however, the DPPH test and β-carotene bleaching tests did not confirm these results. The presented findings may be useful for designing nutraceuticals with programmed release of bioactive compounds during digestion.

## 1. Introduction

Fruits belonging to the Rosaceae and Ericaceae families have shown therapeutic potential in age-related neurological disorders, including Alzheimer’s disease (AD), as extensively discussed in excellent reviews, such as those by Norouzkhani et al. [1]. A meta-analysis by Cheng et al. [2] demonstrated the significant role of dietary flavonoids, which are widely present in these families, in maintaining human cognition at an appropriate level. The mean intake of total polyphenols among Polish, American, French, and British adults and elderly individuals aged 45 to 69 years is estimated at 1740.7 ± 630.2, 989.3 ± 360, 1193 ± 510, and 1053.2 ± 545.3 mg/day, respectively, with an overall mean polyphenol intake of 1666.3 ± 41.7 mg/day [2,3,4,5,6]. The lowest mean intake was reported for Brazilian individuals aged 20 to 59 years at 458.8 ± 337.61 mg/day [7]. It appears that the intake of polyphenols is approximately 1–1.5 g/day and likely does not exceed 2 g. However, these compounds are treated as xenobiotics and are deliberately removed from the body. Furthermore, it has been repeatedly noted that the bioavailability of polyphenols from the gastrointestinal tract is low, even after high doses are consumed, and their metabolism resembles the detoxification process of xenobiotics [8,9]. Since consuming high doses of polyphenols is ineffective, a new approach should be developed: small portions consumed several times a day, including just before sleep. This method would replace a single peak of a large increase in blood polyphenol concentration with several smaller but quantitatively sufficient increases throughout the day and night. This strategy would ensure overlapping peaks of polyphenols in the bloodstream, providing continuous protection even long after falling asleep. The effect could be enhanced by encapsulating polyphenols in a food matrix that is resistant to mechanical, chemical, and enzymatic degradation during digestion. Therefore, the aim of this preliminary work was to create a standardized PP derived from seven selected fruits that can support nutritional treatment for AD. The preparation is the result of screening work (dozens of tested parts of plants—fruits, vegetables, herbs) aimed at creating a complementary mixture of many polyphenolic compounds. As the most important tested activity of the created PP, we have chosen the preventive effect on AD, due to the strong anti-acetyl- and anti-butyrylcholinesterase, antioxidant, reducing, and anti-inflammatory activities. Thirteen gelling agents were tested to produce jellies containing PP, and gelatin was chosen to create a three-layer jelly. Measurements were taken to assess the release of bioactive compounds from the jelly and the evolution of selected biochemical activities during in vitro digestion.

## 2. Materials and Methods

### 2.1. Materials

Fruits (peach *Prunus persica* L., apricot *P. armeniaca* L., chokeberry *Aronia melanocarpa* L., cranberry *V. oxycoccus* L., raspberry *Rubus idaeus* L., and wild strawberry *Fragaria vesca* L.) were gathered from locations as described in Szwajgier et al. [10] and Gajowniczek-Ałasa et al. [11]. Blueberries (*V. corymbosum* L.) were obtained from the Partnership Wholesale Market S.A. (Elizówka 65, Ciecierzyn, Poland). All fruits were in a state of ripeness for consumption.

Agar (E406), amidated pectin with dextrose (E440ii), carrageenan with xanthan gum (E407), guar gum (E412), pork gelatin (Bloom 180), and xanthan gum (E415) were sourced from Agnex (Białystok, Poland). Cellulose (E460) was obtained from JRS (Patterso, NY, USA). Locust bean gum (carob gum) was sourced from ViVio (Krakow, Poland). Methylcellulose was acquired from ProFood (Naperville, IL, USA). Sodium caseinate (100%) was obtained from TrecNutrition (Seattle, WA, USA). Highly methylated pectin was sourced from Superior-Strefa (Dobre Miasto, Poland). Starch was obtained from ViVio (Poland) and WarChem (Zakręt, Poland).

Standards of phenolic compounds were of HPLC purity (detailed information available upon request). All other reagents were sourced from Avantor (formerly P.O.Ch, Gliwice, Poland).

### 2.2. Producion of Polyphenolic Preparation (PP)

A portion of 1000 g of each fruit was crushed while being heated simultaneously (Thermomix TM31, Vorwerk, Wuppertal, Germany, at half speed) to 80–90 °C for 5 min, then cooled to 50–55 °C and further crushed for 3 min at maximum speed. To each fruit portion, hydrolytic enzyme preparations were added at doses recommended by the manufacturers: Panzym Flux (3 mL/100 dm^3^), Rohapect UF (20 ppm), Rohament CL (5 mg/100 g), Rohalase BXL (20 g/100 L), Rohapect PTE (20 ppm), Gammadex CAL (15 mg/dm^3^), and Panzym Pro Color (1 mL/10 kg). Pectinex BE XXL was added at a concentration of 0.3 mL/kg according to Narwojsz and Borowska [12]. The samples were incubated at 45 °C for 2 h. Solid components from each fruit preparation were separated by centrifugation (30 min at 4 °C, 9000× *g*), and each juice was directly microfiltered (PES 0.2 µm, VF05P7) followed by ultrafiltration (PES 5 kDa, VF05P1, Vivaflow 50 system, Sartorius Stedim, Aubagne, France, at room temperature). Each ultrafiltrate was concentrated to achieve a Brix level of 40–70 (−0.09 MPa, 40 °C). Just before mixing, to address troublesome precipitations, each ultrafiltrate was separately centrifuged again (30 min at 4 °C, 9000× *g*), and the final herein studied PP was obtained by mixing 7.5 parts (by mass) of the concentrated peach ultrafiltrate with 0.3 parts (by mass) of each of the other ultrafiltrates.

### 2.3. Dry Mass

The dry mass of all samples was determined as previously described [11].

To evaluate the total polyphenol content (TPC), individual classes of polyphenols, and in vitro biological activities (anti-cholinesterase, anti-cyclooxygenase-2, anti-catalase, superoxide dismutase, glutathione peroxidase, glutathione reductase activities, ferric reducing antioxidant power, cupric reducing antioxidant capacity, hydroxyl radical antioxidant capacity, oxidation of linoleic acid, β-carotene bleaching test, ABTS, and DPPH antiradical activities), the dry matter contents in PP and individual ultrafiltrates from fruit juices were standardized by dissolving the ultrafiltrates (54.26 mg of peach, 57.11 mg of raspberry, 54.69 mg of blueberry, 50.27 mg of cranberry, 51.20 mg of wild strawberry, 50.80 mg of apricot, 49.62 mg of chokeberry, and 54.45 mg of PP) in 1.5 mL of distilled water.

### 2.4. Total Phenolic Content (TPC)

The Folin–Ciocalteu method was employed following the modification by Studzińska-Sroka [13], using the following volumes of reagents: 15 μL of the sample, 50 μL of distilled water, and 750 μL of Folin–Ciocalteu reagent. After 5 min, 750 μL of a 7.5% Na_2_CO_3_ solution was added. A calibration curve was prepared using 16 solutions of gallic acid (28.8–480 μg gallic acid/mL) as described above. Each sample was analyzed in triplicate.

### 2.5. Total Flavonoid Content (TFvC)

The method used was based on Szwajgier et al. [14] with slightly modified volumes of the reagents: 20 µL of the sample, 0.3 mL of an 80% (*v*/*v*) ethanol solution, 10 µL of a 10% (*v*/*v*) Al(NO_3_)_3_ solution, and 10 µL of a 1 M potassium acetate solution. A calibration curve (y = 2.3219x) was prepared using a stock solution of 0.25 mg of quercetin (dissolved in 0.25 mL of deionized water and 0.25 mL of acetonitrile), which was then diluted to create 12 working solutions. Absorbance was measured using a Tecan Sunrise microplate reader (Grödig, Austria). Each sample was analyzed in triplicate.

### 2.6. Total Flavanol Content (TFnC)

The method used was based on Szwajgier et al. [14] with slightly modified volumes of reagents: 20 µL of the sample and 208 µL of 1,4 mL p-dimethylaminocinnamaldehyde (Sigma D4506, St. Louis, MO, USA). TFnC was calculated using a calibration curve (y = 15.702x − 1.4132) prepared from 18 dilutions of a stock catechin solution (3.25 mg of (+)-catechin dissolved in 1.87 mL of methanol). Absorbance was measured using a Varioskan Lux (Thermo Scientific, Carlsbad, CA, USA) microplate reader. Each sample was analyzed in triplicate.

### 2.7. Total Condensed Tannins (TCT)

The method used was based on Szwajgier et al. [14] with slightly modified volumes of reagents: 14 µL of the sample and 0.25 mL of a 4% vanillin solution. The TCT content was calculated using a calibration curve (y = 15.702x − 1.4132) prepared from 15 dilutions of a stock (+)-catechin solution (3.45 mg of catechin dissolved in 2 mL of methanol). Absorbance was measured using a Tecan Sunrise microplate reader (Grödig, Austria). Each sample was analyzed in quadruplicate.

### 2.8. Total Anthocyanin ContenT (TAC)

The method used was based on Szwajgier et al. [14] with slightly modified volumes of the reagents: 50 µL of the sample and 208 µL of a 0.4 M acetate buffer (pH 4.5) or HCl/KCl buffer (pH 1.0). Absorbance was measured using a Varioskan Lux (Thermo Scientific) microplate reader. Each sample was analyzed in quadruplicate.

### 2.9. Qualitative and Quantitative LC-MS (HPLC-HRMS and HRMS/MS)

HPLC with UV and spectrofluorimetric detection was performed as described in detail in the Supplementary Materials. HPLC-HRMS and HRMS/MS analyses were conducted as outlined in Szwajgier et al. [10].

### 2.10. Selecting of the Final Gelling Agent

To select the final gelling agent, various gelling agents were tested (Table S4), following the provided instructions as well as those by Gustaw and Mleko [15], Baryłko-Pikielna and Matuszewska [16], Cebi et al. [17], and de Moura et al. [18]. Jellies (8.5–9.7 g, depending on the gelling agent) were produced using rectangular silicone molds (Figure S9). The jellies were digested in vitro, and the overall significant disintegration of the jellies was evaluated. Based on the results, pork gelatin was chosen as the gelling agent due to its high resistance to disintegration (Table S5). The concentration of gelatin in the prototype was further refined, and the final jelly recipe is presented below.

### 2.11. Final Jelly Recipe

Final jelly was produced using PP produced as described in Section 2.2. **Middle Layer with PP:** 12 g of gelatin was dissolved in 20 mL of water at 70–80 °C. A volume of 7 mL of the gelatin solution was mixed with 7.5 g of concentrated peach ultrafiltrate and 0.3 g of each of the remaining ultrafiltrates (all previously centrifuged for 30 min at 9000× *g*).

**Outer Layers of the Jelly:** 12 g of gelatin was dissolved in 20 mL of water at 70–80 °C. A volume of 7 mL of this gelatin solution was thoroughly mixed either with 7.5 g of concentrated apricot or peach ultrafiltrate so one outer layer was produced using apricot preparation and one using a peach one.

**Formation of the Jelly:** Rectangular silicone molds were used to produce jellies (9.5 g, Figure S9). The first outer layer (peach) was poured to a thickness of 4 mm (3 g in total) and placed at 4–8 °C to set. Next, the middle layer containing PP (4 mm thick, 3.5 g) was poured and also placed at 4–8 °C to set. Finally, the second outer layer (apricot, 4 mm thick, 3 g) was poured and placed at 4–8 °C to set. The jellies were stored for 8 h at −4 °C in a closed package.

### 2.12. Consumer Evaluation of the Jellies—A Pilot Study

This pilot analysis involved 16 consumers (5 men and 11 women, aged 20–65 years) who correctly recognized sweet, salty, sour, and bitter tastes and demonstrated appropriate thresholds for taste perception and taste difference detection [19]. All participants regularly consumed jellies (at least several times a month) and were briefly instructed on the characteristic features and compositions of fruit jellies, as well as on common defects found in this type of product. A form was created using a 9-point hedonic scale for the descriptive test, based on original sources [20,21,22,23,24,25]. Samples were assessed anonymously in a well-ventilated room (20–22 °C), free from odors and sunlight, and placed on transparent glass Petri dishes on snow-white sheets of paper, with access to still water. The following options were available: 9—extremely positive feeling; 8—very positive feeling; 7—moderately positive feeling; 6—slightly positive feeling; 5—neither positive nor negative feeling; 4—slightly negative feeling; 3—moderately negative feeling; 2—very negative feeling; 1—extremely negative feeling. Commercial jellies were evaluated simultaneously. The product analysis was conducted twice. The results presented in the table are shown on a conventional scale [16], with the arithmetic mean displayed in the graphs.

### 2.13. In Vitro Digestion

In vitro “digestions” of the polyphenolic preparation (PP, produced as described in Section 2.2) and jellies (produced in Section 2.11) were carried out using the method described by Baranowska-Wójcik et al. [26] with modifications outlined below. The equipment used is detailed in Baranowska-Wójcik et al. [26] (Figure S10). A portion of two jellies, cut with a scalpel in a standardized manner (8 × 5 × 3 mm), or a mixture of corresponding portions of liquid PP and concentrated ultrafiltrates from peach and apricot (3.87 g + 3 g + 3 g, respectively), were mixed in a “digestive” tank with 35 mL of distilled water and 35 mL of simulated salivary fluid. Next, 0.25 mL of a 0.3 mol/L CaCl_2_ solution (Sigma 746495), 9.75 mL of distilled water, and 2 mL of an α-amylase solution containing 451 enzyme units (human saliva, Sigma-Aldrich A0521) were added. The samples were kept at 37 °C while stirring at 50 rpm for 2 min. Then, 60 mL of simulated gastric fluid (SGF) and 0.04 mL of the 0.3 mol/L CaCl_2_ solution were added; the pH was adjusted to 2.5 with HCl solution (Sigma H1758, for molecular biology), and pepsin solution (40 mg, Sigma P6887, dissolved in 6.4 mL of SGF) was added. A sample of 20 mL was taken for analysis to mark the start of the gastric phase. After 10 min, a second portion of pepsin (40 mg in 6.4 mL of SGF) was added. Stirring was reduced to 10 rpm, and samples (20 mL each time) were taken after 10 and 20 min to mark the end of the gastric phase. Next, 44 mL of simulated intestinal fluid (SIF), along with 1.224 g of pancreatin (from porcine pancreas, Sigma P1625) and 7.33 g of bile salts (Sigma 48305)—both suspended in 37.5 mL of SIF—were added (the amounts for pancreatin and bile salts were calculated according to Steward et al. [27]. Then, 0.2 mL of the 0.3 mol/L CaCl_2_ solution was added, the pH was adjusted to 7.0 using a 1 mol/L NaOH solution, and the “intestinal” volume was brought up to 161.7 mL with distilled water. Samples (20 mL) were taken every 20 min from 0 to 100 min to mark the end of the intestinal phase. Simultaneously, two reagent (blank) samples were run, which were subtracted during calculations: one sample without jellies or liquid ultrafiltrates and another sample using jellies produced without added concentrated ultrafiltrates. All “digestions” were performed in duplicate. During the entire “digestion”, sterile CO_2_ (analytical grade, Linde Gas Poland (Lublin, Poland), approximately 5–8 mL/min, PTFE syringe filter 0.45 µm) was passed through each “digested” sample individually. Immediately after collection, each sample was analyzed according to the methods described below or stored at −80 °C until analysis.

### 2.14. In Vitro Biological Activities

#### 2.14.1. Effect on Acetyl- and Butyrylcholinesterase (Anti-AChE and Anti-BChE Activity)

The analysis was performed as described in Studzińska-Sroka et al. [13], except that the volume of the tested sample was 10 µL (for PP and individual concentrated ultrafiltrates) or 140 µL (for the in vitro digested sample, replacing an equal volume of Tris buffer). Each sample was analyzed in quadruplicate.

#### 2.14.2. Effect on Cyclooxygenase-2 (COX-2) Activity

The method described in Studzińska-Sroka et al. [13] was used with the following modifications: the volume of the tested sample was 10 µL (for PP and individual concentrated ultrafiltrates) or 120 µL (for in vitro digested samples, replacing an equal volume of Tris buffer). Each sample was analyzed in duplicate.

#### 2.14.3. Effect on Catalase (CAT) Activity

Analysis was performed as described in Watanabe et al. [28] with modified volumes of reagents: 900 µL of EDTA (Sigma E-9884) solution, 20 µL of the sample (PP and individual concentrated ultrafiltrates) or 140 µL of the in vitro digested sample, replacing an equal volume of buffer, 1 µL of catalase (Sigma C3515, 20-fold diluted), 45 µL of 1 M Tris-HCl (pH 7, Sigma 93363), and 4 µL of 30% H_2_O_2_ (Sigma H1009). Each sample was analyzed in quadruplicate.

#### 2.14.4. Effect on Superoxide Dismutase (SOD) Activity

The method used was based on Szwajgier et al. [14] with slightly modified volumes of reagents: the sample (20 µL of PP and individual concentrated ultrafiltrates or 120 µL of in vitro digested sample, replacing the same volume of buffer to achieve the final volume of the mixture as in the cited method) was mixed with 10 µL of SOD solution (0.6 U, Sigma S5395), 80 µL of nitroblue tetrazolium solution (Sigma N6639), 110 µL of phosphate buffer (Sigma 60218 and 60353), 15 µL of xanthine (Sigma X0626), and 4 µL of xanthine oxidase (Sigma X4875). Each sample was analyzed in quadruplicate.

#### 2.14.5. Effect on Glutathione Peroxidase (GPx) and Glutathione Reductase (GR) Activity

Both analyses were performed as described in Studzińska-Sroka et al. [13], but the volume of the tested sample was 20 µL (for PP and individual concentrated ultrafiltrates) or 150 µL (for the in vitro digested sample, replacing an equal volume of buffer to achieve the final volume of the mixture as specified in the cited method). Each sample was analyzed in quadruplicate.

#### 2.14.6. Ferric Reducing Antioxidant Power (FRAP)

The method used was based on Szwajgier et al. [14] without changes, except that the volume of the FRAP solution was 380 μL and the sample volume was 20 μL (for PP and individual concentrated ultrafiltrates) or 150 μL for the in vitro digested sample (replacing an equal volume of buffer to achieve the final volume of the mixture as specified in the cited method). A calibration curve (y = 0.0796) was prepared using 20 standard solutions (0.0255–0.51 mg of Trolox/mL) from a stock Trolox solution. Each sample was analyzed in quadruplicate.

#### 2.14.7. Cupric Reducing Antioxidant Capacity (CUPRAC)

The method described in Szwajgier et al. [14] was used, but the volume of the tested sample was 20 μL of PP and individual concentrated ultrafiltrates or 150 μL of the in vitro digested sample, along with 180 μL of ammonium acetate buffer (pH 7.0, 1 M), 110 μL of neocuproine solution (Sigma N1501, 3.75 mM, dissolved in DDI water:ACN 1:1), and 80 μL of 10 mM CuCl_2_ (Sigma 307483). Each sample was analyzed in quadruplicate.

#### 2.14.8. Hydroxyl Radical Antioxidant Capacity (HORAC)

The analysis was performed as described in Szwajgier et al. [14] with a slightly modified volume of the tested sample (10 μL of PP or individual concentrated ultrafiltrates, or 100 µL of the in vitro digested sample). Activity was expressed in gallic acid equivalents, using 11 gallic acid solutions ranging from 8.1 to 350 μg/mL. Each sample was analyzed in quadruplicate.

#### 2.14.9. Linoleic Acid Oxidation Test

The method of Szwajgier et al. [14] was used. Each sample was analyzed in quadruplicate.

#### 2.14.10. Beta-Carotene Bleaching Test

The method described in Szwajgier et al. [14] was used; however, the β-carotene/linoleic acid emulsion (Sigma C9750/L-1376) was prepared using modified proportions of the components: 7 mg of β-carotene with 5 mL of chloroform, 350 μL of linoleic acid, and 2.8 g of Tween 80. Each sample was analyzed in quadruplicate.

#### 2.14.11. Antiradical Activity (ABTS*^+^)

The method of Gajowniczek-Ałasa et al. [11] was used, but the volume of the tested sample was 20 μL (for PP or individual concentrated ultrafiltrates) or 150 µL for the in vitro digested sample (replacing an equal volume of distilled water to achieve the final volume of the mixture as specified in the cited method). For the calibration curve (y = 20.171x + 0.822), 15 Trolox solutions (water/ethanol 1:1 *v*/*v*; 0.6–0.03 mmol/L) were used. Each sample was analyzed in quadruplicate.

#### 2.14.12. Antiradical Activity (DPPH)

The method of Gajowniczek-Ałasa et al. [11] was used, but the volumes were as follows: 20 μL (for PP and individual concentrated ultrafiltrates) or 150 μL of the in vitro digested sample (which replaced the same volume of methanol), 30 μL of DPPH solution, and methanol (Fluka 34966, Buchs, Switzerland). For the calibration curve (y = 18.823x + 0.443), 15 Trolox solutions (water/ethanol 1:1 *v*/*v*; 0.6–0.03 mmol/L) were used. All samples were analyzed in quadruplicate.

### 2.15. Statistical Analysis

The obtained data were expressed as mean (± standard error of the mean (SEM)). Statistically significant differences were calculated using Tukey’s HSD test (STATISTICA 13.0, StatSoft, Kraków, Poland) with significant differences identified at *p* < 0.05, and all results presented in a given figure were compared side by side.

## 3. Results

As can be seen in Table 1, TPC of PP was 8.10 ± 0.45 mg GAE/g, resulting from the mixing of all the ultrafiltrates included in the mixture (PP was obtained by combining 7.5 parts by mass of concentrated peach ultrafiltrate with 0.3 parts by mass of all other fruit ultrafiltrates, as detailed in Section 2.11). The highest contribution to the PP was attributed to TCT, which measured 3.54 ± 0.22 mg catechin/g, followed by TFvC at 1.57 ± 0.03 mg quercetin/g. Among the individual compounds identified by LC-MS (Table 2), the analysis confirmed the presence of 26 main polyphenols, including flavonoids such as flavonols (quercetin and its derivatives, kaempferol, kaempferol 3-rutinoside), flavan-3-ols (epicatechin), anthocyanins (cyanidin, delphinidin, pelargonidin, cyanidin-3-glucoside, and pelargonidin 3-*O*-glucoside), and proanthocyanidins like procyanidin B2. A significant portion of the identified polyphenolic compounds consisted of phenolic acids, totaling 2102.3 μg/g out of 7450.6 μg/g of all individual compounds identified in PP (Table 2). Additionally, ellagic acid and dihydrochalcone phloridzin were also present.

### 3.1. In Vitro Biological Activities of Inividual Ultrafiltrates and PP

PP demonstrated strong anti-AChE and anti-BChE activities (Table 3). Additionally, all individual ultrafiltrates and PP exhibited anti-CAT, anti-SOD, anti-GPx, and anti-GR activities. Last but not least, PP effectively decreased COX-2 activity by 57.0 ± 4.4% under the conditions applied in this study.

The antioxidant activities tested in this study demonstrated a positive effect for each individual ultrafiltrate as well as for the final PP across all methods applied (Table 4). The observed activity varied depending on the method used. Therefore, a comparison of eight related samples (which differ in terms of plant species) using multiple methods should be considered essential for accurately measuring activity. The absence of such a comparison among methods that differ in their mechanisms of action can sometimes lead to erroneous conclusions regarding the comparison of the samples under investigation.

### 3.2. Consumer Analysis of Jellies

**Figure 1:**
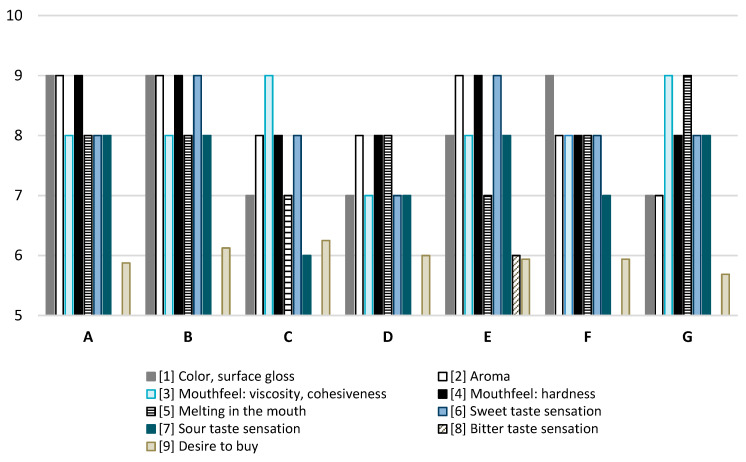
Consumer analysis of jellies. A—jelly designed in this study. B–G commercial jelly beans available on the market.

The studied jellies (A) ranked highest among all tested jellies in terms of color and surface reflection, aroma, and firmness (Figure 1). In terms of viscosity and cohesion, only samples C and G received higher scores. Regarding melting in the mouth, only sample G achieved higher scores. The perception of sweet taste was higher in samples B and E compared to sample A, while the sour and bitter tastes of the tested sample were comparable to those of the other jellies. Consumers expressed a willingness to purchase the jellies we created at a level comparable to, or even higher than, their willingness to purchase other commercial jellies with which they were compared.

### 3.3. Changes in the Content of Polyphenolic Compounds in PP and PP-Enriched Jellies During In Vitro Digestion

When discussing the effect of in vitro digestion on the release of bioactive compounds, as well as the impact on enzymes and the biological properties of PP, two aspects should be considered: the effect of entrapment of PP in the jelly compared to liquid PP, and the evolution of properties during the successive stages of digestion.

**Figure 2:**
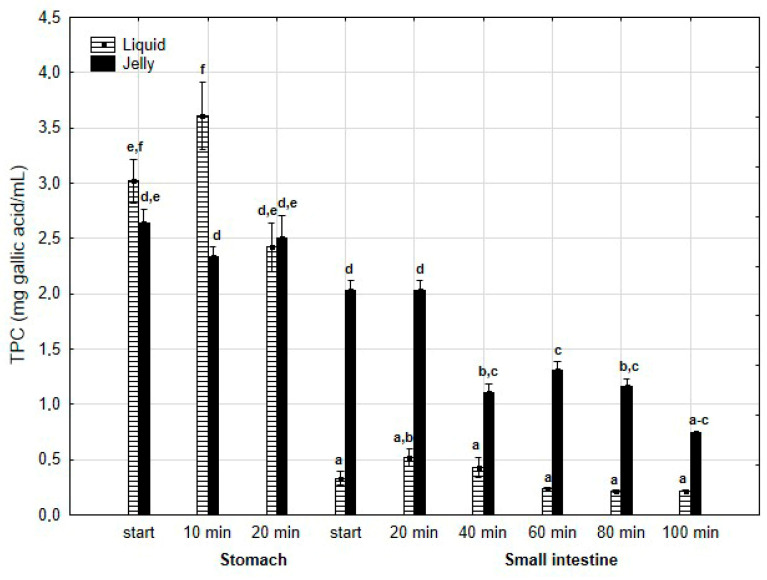
Evolution of TPC during in vitro digestion. Different letters represent statistical differences at *p* < 0.05. All results presented in a given figure were compared side by side.

**Figure 3:**
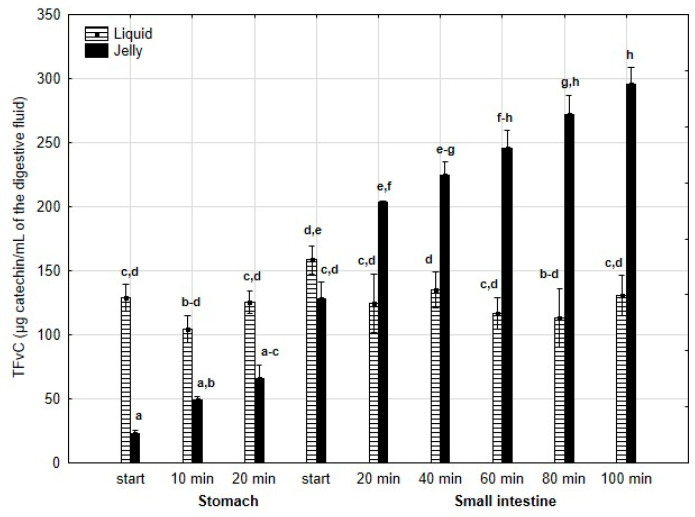
Evolution of TFvC during in vitro digestion. Different letters represent statistical differences at *p* < 0.05. All results presented in a given figure were compared side by side.

TPC (Figure 2) was higher (although mostly statistically insignificant) in the “digested” fluid at the “stomach” stage when applied in liquid form. However, throughout the entire “intestinal” phase, TPC was significantly (*p* < 0.05) higher in the “digested” fluid containing PP entrapped in the jelly. In the case of liquid PP, TPC decreased drastically at the beginning of the “small intestine” and remained low, with no statistically significant changes, until the end of the “intestine”. In contrast, when applied in jelly form, TPC gradually decreased until the end of the “intestine”. TFvC at the “stomach” stage was higher when liquid PP was applied (although mostly statistically insignificant, Figure 3). However, starting from 20 min into the “intestinal” digestion, TFvC was significantly (*p* < 0.05) higher in the “digested” liquid containing jelly with PP. In the case of the “digested” jelly, TFvC increased gradually until the end of the “intestine”.

**Figure 4:**
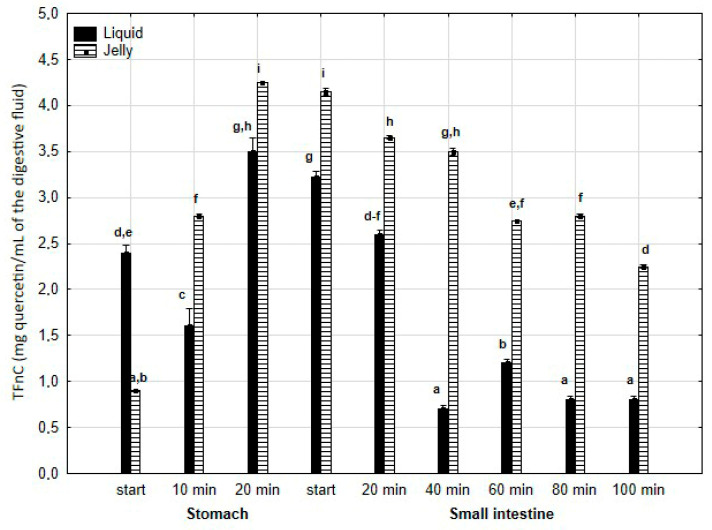
Evolution of TFnC during in vitro digestion. Different letters represent statistical differences at *p* < 0.05. All results presented in a given figure were compared side by side.

TFvC (Figure 4) was significantly (*p* < 0.05) higher after 10 min of “digestion” at the “stomach” stage and continued to be elevated until the end of the “small intestine” stage when PP was applied in the form of jelly. In the case of the jelly with entrapped PP, TFvC gradually and significantly (*p* < 0.05) decreased from the start of the “intestinal” phase until the end of digestion. In contrast, liquid PP lost TFvC after 40 min of “intestinal digestion”, followed by stabilization of the content.

**Figure 5:**
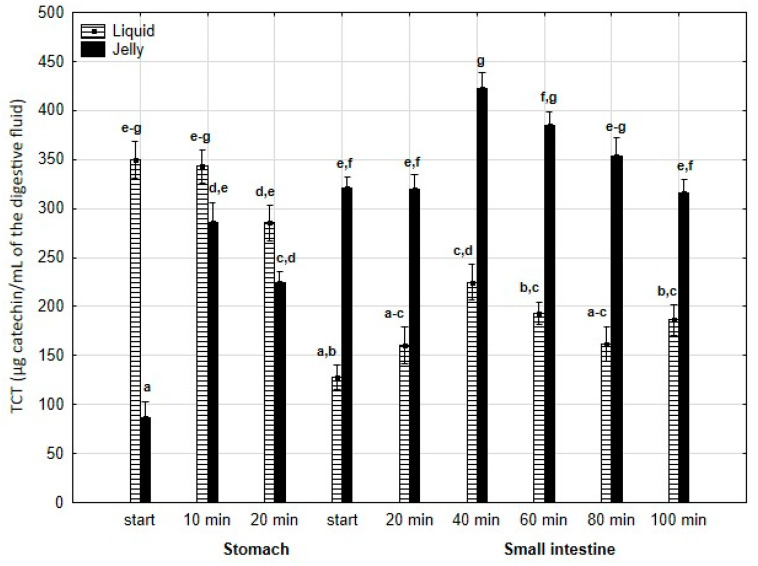
Evolution of TCT during in vitro digestion. Different letters represent statistical differences at *p* < 0.05. All results presented in a given figure were compared side by side.

TCT (Figure 5) was significantly (*p* < 0.05) higher at the beginning of digestion during the “stomach” phase when liquid PP was used; however, by the end of the gastric stage, the differences between the two forms of PP remained statistically insignificant. During the first 40 min of the “intestinal” phase, an increase in TCT was observed for both the liquid and jelly forms of PP, followed by a decrease in TCT until the end of digestion. Notably, at every stage of “intestinal” digestion, TCT content in the “digested” fluid containing jelly was significantly (*p* < 0.05) higher than TCT content in the “digested” fluid containing liquid PP.

**Figure 6:**
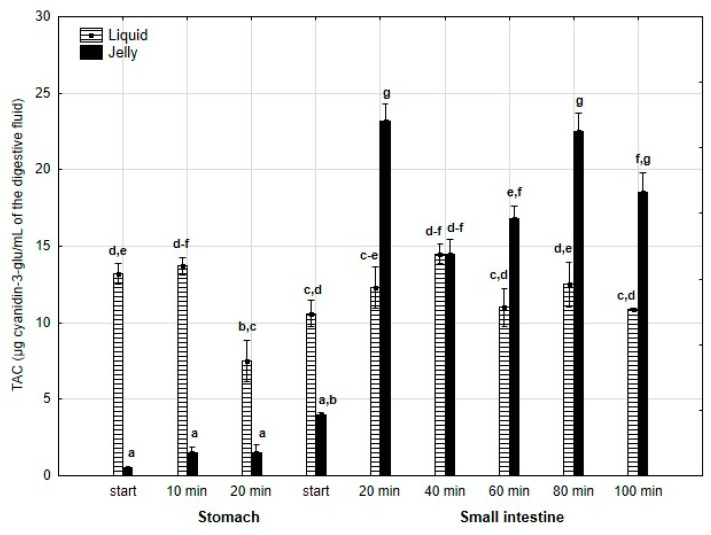
Evolution of TAC during in vitro digestion. Different letters represent statistical differences at *p* < 0.05. All results presented in a given figure were compared side by side.

Last but not least, TAC (Figure 6) was significantly (*p* < 0.05) higher throughout the entire “stomach” phase and at the beginning of the “intestinal” phase when liquid PP was used. However, in the later stages of “intestinal” digestion, TAC in the “digested” fluid containing jelly was significantly (*p* < 0.05) higher than that in the “digested” fluid containing liquid PP. In both forms of PP, stabilization of TAC was observed until the end of digestion.

**Figure 7:**
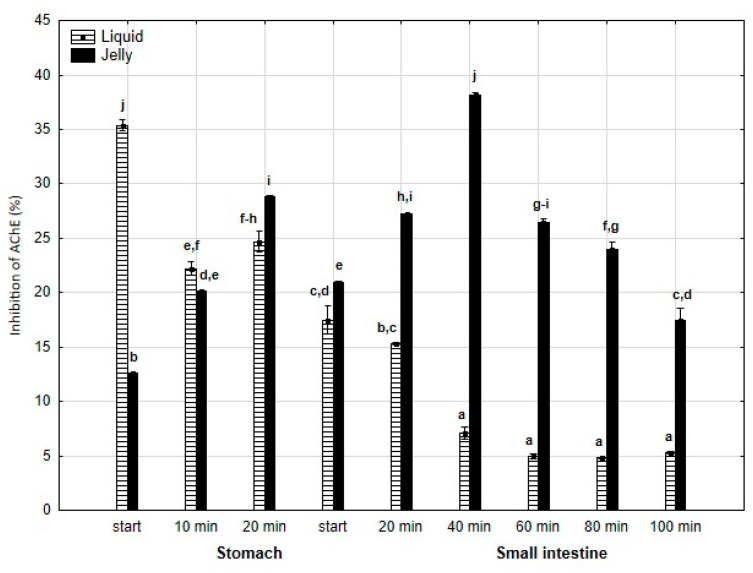
Evolution of anti-AChE activity of PP during in vitro digestion. Different letters represent statistical differences at *p* < 0.05. All results presented in a given figure were compared side by side.

Anti-AChE activity (Figure 7) was significantly (*p* < 0.05) higher at the beginning of the “stomach” phase when liquid PP was used; however, starting from the end of the gastric stage, the inhibitory activities were reversed (*p* < 0.05). In the case of the jelly with entrapped PP, the activity gradually and significantly (*p* < 0.05) increased until 40 min of “intestinal” digestion, followed by a gradual and significant decrease until the end of digestion. In contrast, liquid PP dramatically lost its activity after 40 min of “intestinal digestion”, followed by stabilization.

**Figure 8:**
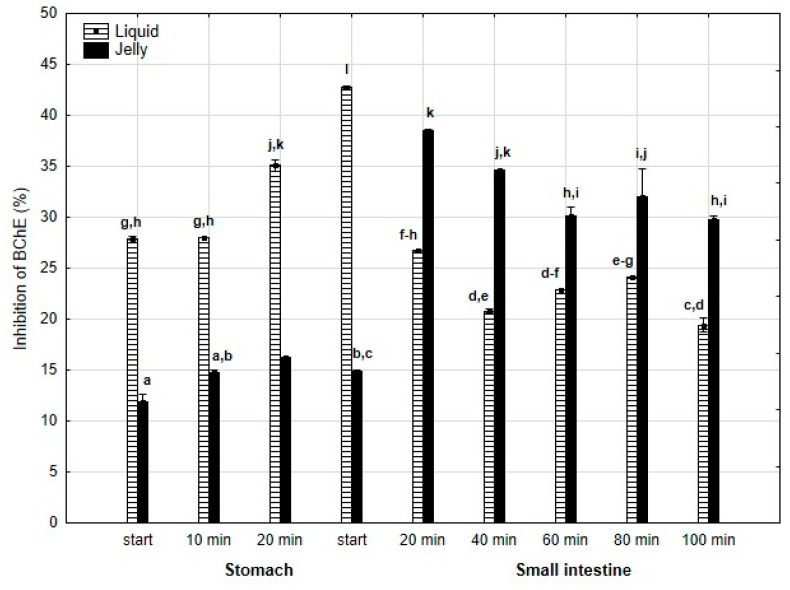
Evolution of anti-BChE activity of PP during in vitro digestion. Different letters represent statistical differences at *p* < 0.05. All results presented in a given figure were compared side by side.

Anti-BChE activity (Figure 8) was significantly (*p* < 0.05) higher throughout the entire “stomach” phase and at the beginning of “intestinal digestion” when liquid PP was used. However, during all later stages of digestion, the anti-BChE activity of the “digested” fluid containing jelly was significantly (*p* < 0.05) higher than that of the fluid containing liquid PP. In the case of the jelly with entrapped PP, the activity gradually and insignificantly (*p* > 0.05) decreased starting from 40 min of “intestinal” digestion until the end of digestion, whereas liquid PP showed stabilization of its activity during the “intestinal” phase.

**Figure 9:**
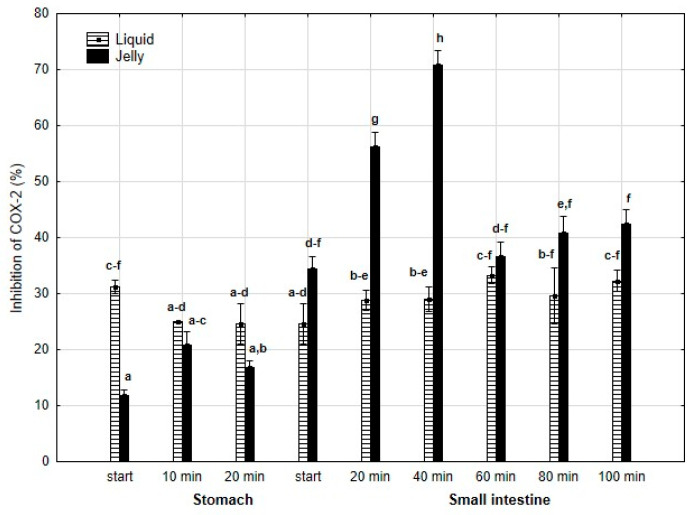
Evolution of anti-COX-2 activity of PP during in vitro digestion. Different letters represent statistical differences at *p* < 0.05. All results presented in a given figure were compared side by side.

Anti-COX-2 activity (Figure 9) was significantly (*p* < 0.05) higher at the beginning of the “stomach” phase when liquid PP was used. However, until the start of the “intestinal” phase, the differences between the two forms of PP remained statistically insignificant. After 20 min of “intestinal digestion”, the anti-COX-2 activity of the “digested” fluid containing jelly was significantly (*p* < 0.05) higher than that of the “digested” fluid containing liquid PP. From this point onward, both forms of PP exhibited stabilized activities, and the differences between them became statistically insignificant again, although the PP entrapped in jelly demonstrated higher anti-COX-2 activity than liquid PP.

**Figure 10:**
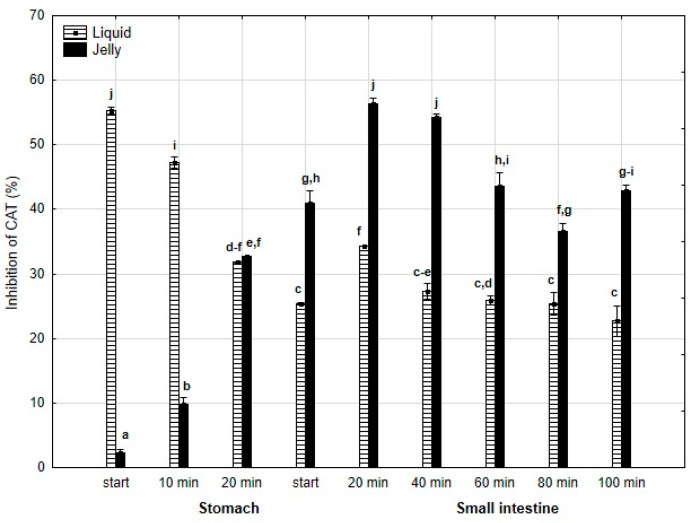
Evolution of anti-CAT activity of PP during in vitro digestion. Different letters represent statistical differences at *p* < 0.05. All results presented in a given figure were compared side by side.

A similar pattern was observed for anti-CAT activity (Figure 10): significantly (*p* < 0.05) higher activity was noted during the “stomach” phase when liquid PP was “digested” compared to PP entrapped in jelly, with equal activity for both forms of PP at the end of the “gastric” phase. Starting from the beginning of the “intestinal” phase and continuing until the end of this phase, the anti-CAT activity of the “digested” fluid containing jelly was significantly (*p* < 0.05) higher than that of the fluid containing liquid PP. From 40 min into the “intestinal” phase, both forms of PP generally exhibited stabilized activities.

**Figure 11:**
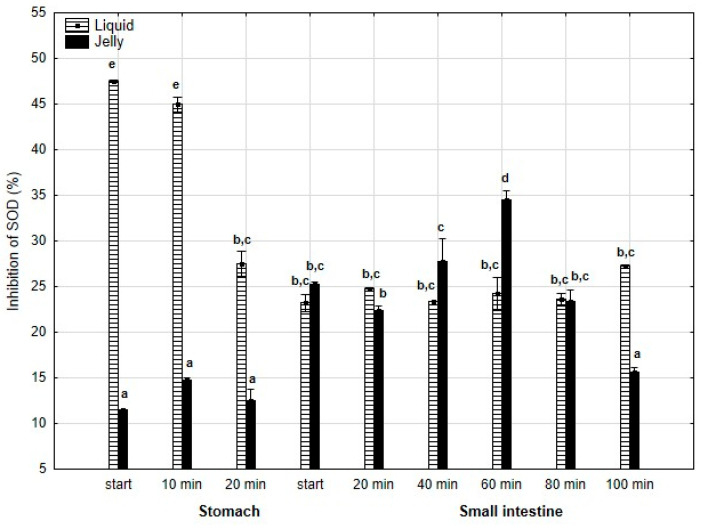
Evolution of anti-SOD activity of PP during in vitro digestion. Different letters represent statistical differences at *p* < 0.05. All results presented in a given figure were compared side by side.

Also, anti-SOD activity (Figure 11) was significantly (*p* < 0.05) higher throughout the entire “stomach” phase when liquid PP was used instead of PP entrapped in jelly. During the initial stages of “intestinal” digestion, the activities of both forms of PP became generally equal until the end of digestion, with a significantly higher activity of PP in jelly form observed at the very end of digestion.

**Figure 12:**
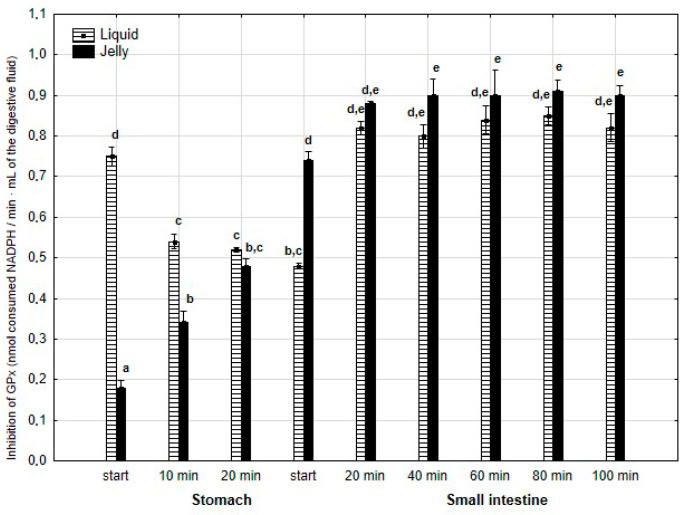
Evolution of anti-GPx activity of PP during in vitro digestion. Different letters represent statistical differences at *p* < 0.05. All results presented in a given figure were compared side by side.

The anti-GPx activity (Figure 12) was significantly (*p* < 0.05) higher during the first 20 min of the “stomach” phase when liquid PP was used instead of PP entrapped in jelly. During the first stage of “intestinal” digestion, the activity of PP in jelly form significantly (*p* < 0.05) exceeded that of liquid PP. Subsequently, the anti-GPx activity of both forms of PP equalized until the end of digestion.

**Figure 13:**
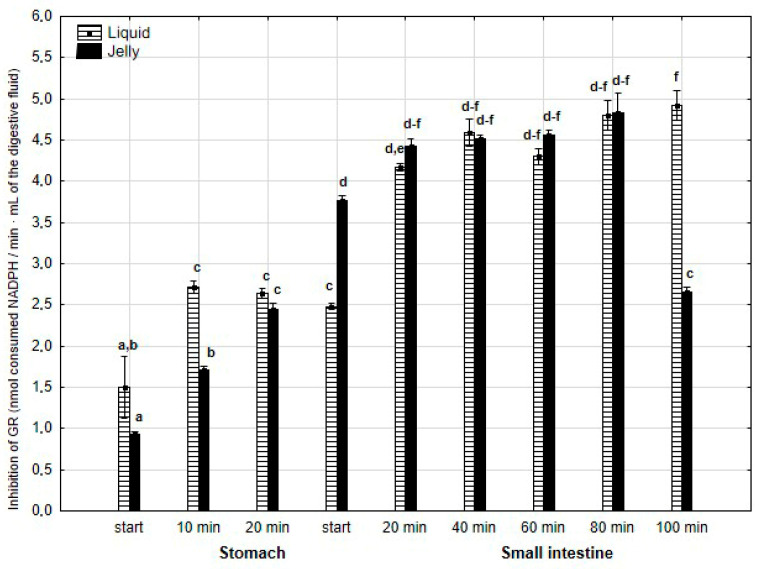
Evolution of anti-GR activity of PP during in vitro digestion. Different letters represent statistical differences at *p* < 0.05. All results presented in a given figure were compared side by side.

The anti-GR activity (Figure 13) was significantly (*p* < 0.05) higher during the first 20 min of the “stomach” phase if the liquid PP was applied for “digestion”, in comparison with PP in the jelly. During the first stage of the “intestinal” “digestion”, the activity of PP in the form of the jelly significantly (*p* < 0.05) exceeded that of the second form of PP. Then, the anti-GPx activity of both forms of PP equalized, with a significantly higher activity of PP in the form of the jelly at a very end of the “digestion”.

**Figure 14:**
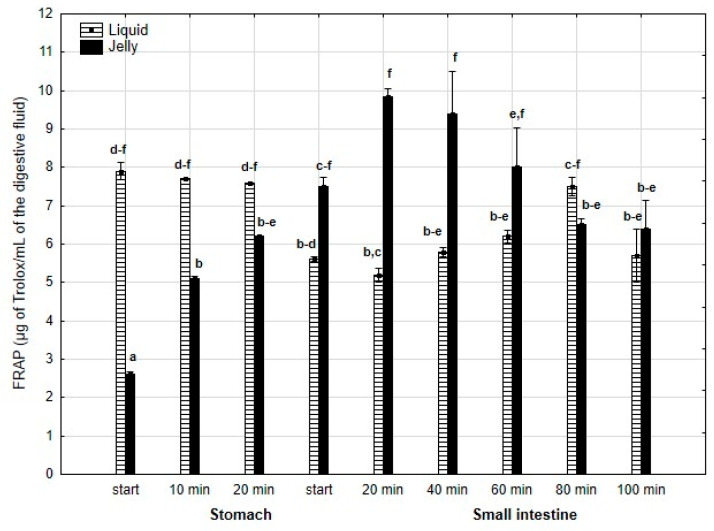
Evolution of FRAP of PP during in vitro digestion. Different letters represent statistical differences at *p* < 0.05. All results presented in a given figure were compared side by side.

FRAP (Figure 14) was significantly (*p* < 0.05) higher during the first 20 min of the “stomach” phase when liquid PP was used for digestion instead of PP trapped in jelly. After 20 min of the “gastric” phase and during the first stage of “intestinal” digestion, the activity of PP in both forms equalized. Subsequently, after 20 min of “intestinal” digestion, the FRAP of the jelly with PP significantly (*p* < 0.05) exceeded that of the liquid form, followed by an equalization of the activity of both forms of PP in the later stages of digestion.

**Figure 15:**
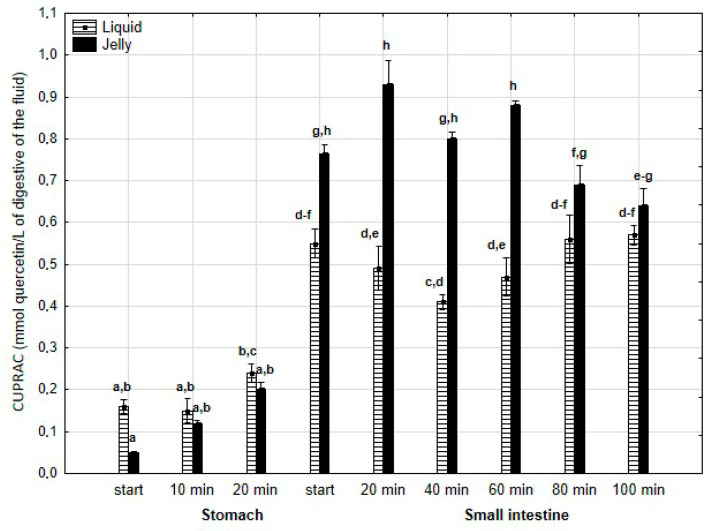
Evolution of CUPRAC of PP during in vitro digestion. Different letters represent statistical differences at *p* < 0.05. All results presented in a given figure were compared side by side.

There was no difference in the CUPRAC activity of both forms of PP during the “stomach” phase (Figure 15). However, the jelly containing PP exhibited higher activity than the liquid form starting from the beginning of the “intestinal” phase and continuing until 80 min into “intestinal” digestion. During the last two measurement times of “intestinal” digestion, the activities of both forms equalized.

**Figure 16:**
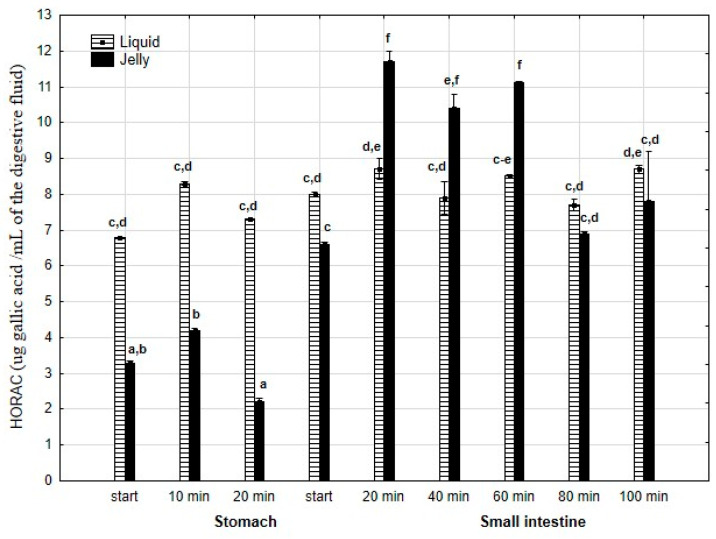
Evolution of HORAC of PP during in vitro digestion. Different letters represent statistical differences at *p* < 0.05. All results presented in a given figure were compared side by side.

HORAC (Figure 16) was significantly (*p* < 0.05) higher throughout the entire “stomach” phase when liquid PP was used for digestion instead of PP trapped in jelly. There was no difference in CUPRAC activity between both forms of PP at the start of the “intestinal” phase. Subsequently, the CUPRAC activity of the jelly containing PP significantly (*p* < 0.05) exceeded that of the liquid form. However, during the last two measurement times of “intestinal” digestion, the activities of both forms equalized.

**Figure 17:**
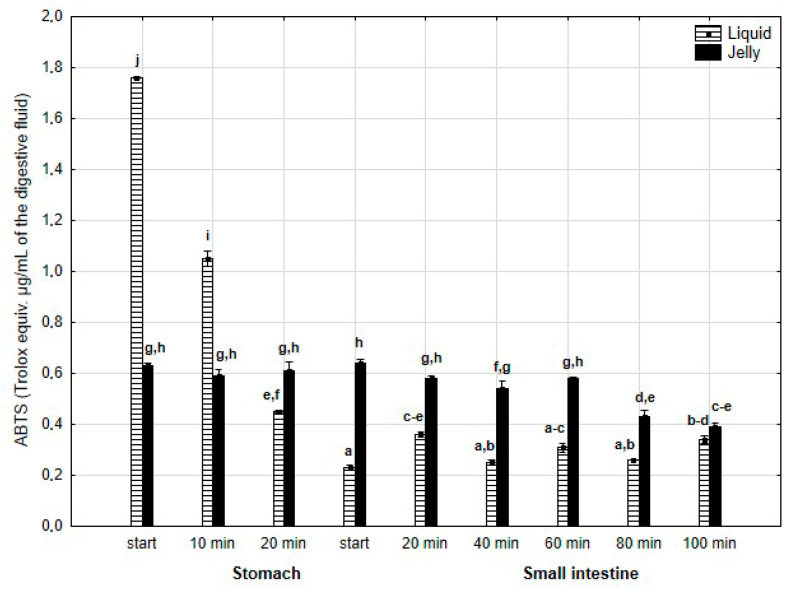
Evolution of the antioxidant activity of PP (with ABTS) during in vitro digestion. Different letters represent statistical differences at *p* < 0.05. All results presented in a given figure were compared side by side.

The antiradical activity (measured with ABTS^+^*, Figure 17) was significantly (*p* < 0.05) higher during the first two measurement times of the “stomach” phase when liquid PP was used for digestion instead of PP trapped in jelly. Subsequently, the activity of the jelly containing PP significantly (*p* < 0.05) exceeded that of the liquid form; however, at the last measurement point of “intestinal” digestion, the activities of both forms equalized.

**Figure 18:**
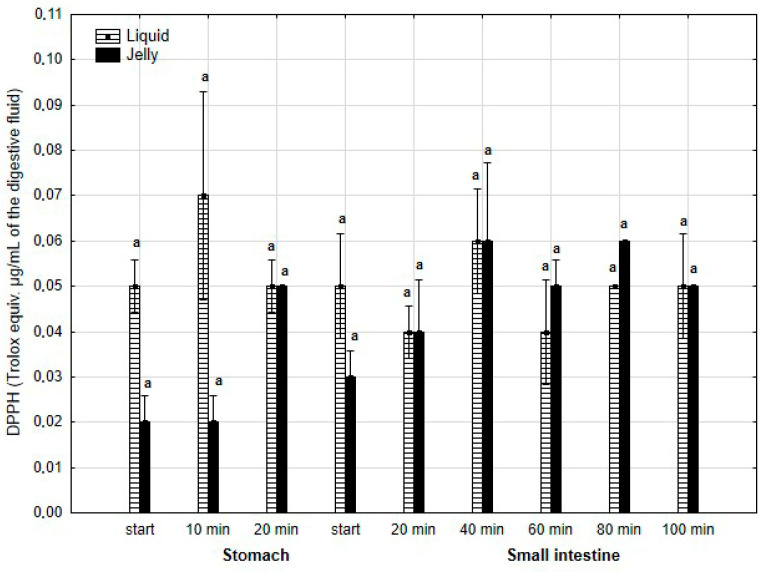
Evolution of the antioxidant activity of PP (with DPPH) during in vitro digestion. Different letters represent statistical differences at *p* < 0.05. All results presented in a given figure were compared side by side.

The antiradical activity (measured with DPPH*, Figure 18) was insignificantly (*p* > 0.05) higher during the first two measurement times of the “stomach” phase when liquid PP was used for digestion instead of PP trapped in jelly. However, during all subsequent stages of digestion, no apparent differences in the antiradical activities of the jelly containing PP and liquid PP were observed.

**Figure 19:**
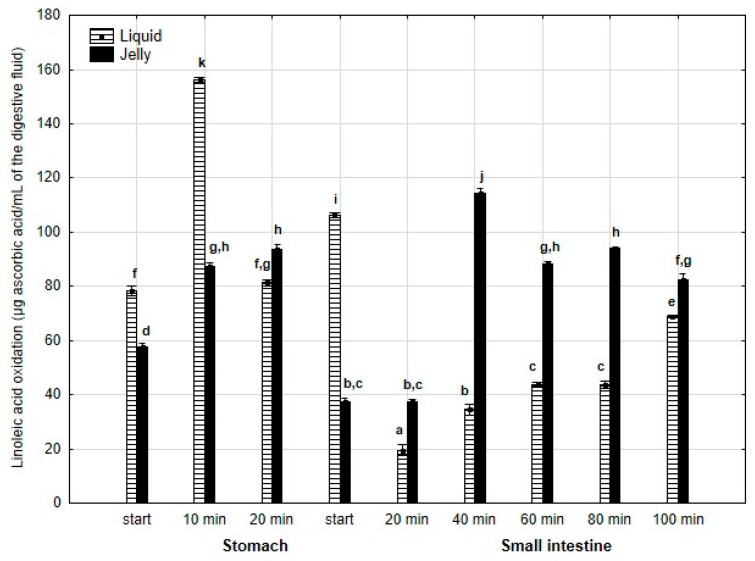
Inhibition of linoleic acid oxidation by PP during in vitro digestion. Different letters represent statistical differences at *p* < 0.05. All results presented in a given figure were compared side by side.

Inhibition of linoleic acid oxidation (Figure 19) was significantly (*p* < 0.05) higher throughout the entire “stomach” phase and at the start of the “intestinal” phase when liquid PP was used for digestion instead of PP trapped in jelly. Subsequently, the activity of the jelly containing PP was significantly (*p* < 0.05) higher than that of the liquid form until the end of digestion. It is noteworthy that the activity of PP entrapped in jelly increased between 20 and 40 min of “intestinal digestion”, followed by stabilization of the activity.

**Figure 20:**
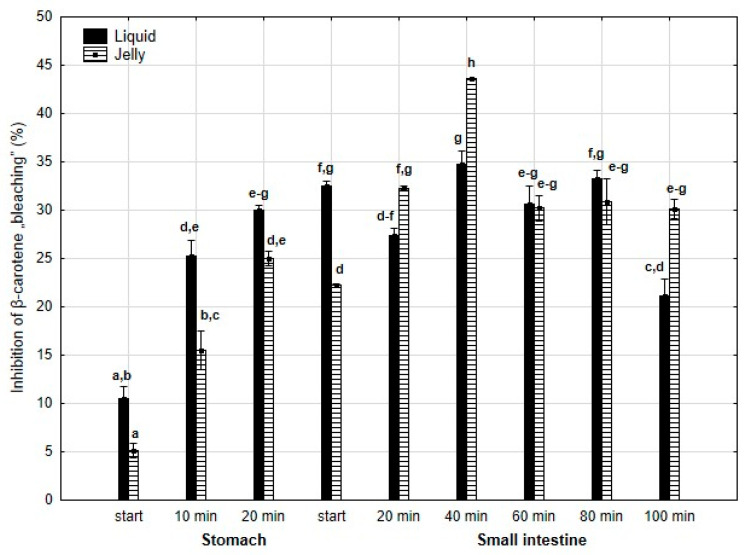
Inhibition of β-carotene “bleaching” by PP during in vitro digestion. Different letters represent statistical differences at *p* < 0.05. All results presented in a given figure were compared side by side.

Inhibition of β-carotene “bleaching” (Figure 20) was higher throughout the entire “stomach” phase and at the start of the “intestinal” phase (either significantly or insignificantly) when liquid PP was used for digestion instead of PP trapped in jelly. Subsequently, the activities of the jelly containing PP and liquid PP largely equalized until the very last measurement point at the end of digestion in the “intestine”, when the activity of PP entrapped in jelly was significantly (*p* < 0.05) higher than that of the liquid form of PP.

## 4. Discussion

The anticholinesterase, antioxidant, and anti-inflammatory activities of the fruits studied in this paper are well established [29,30,31,32,33,34,35]. Indeed, our PP demonstrated strong anti-AChE and anti-BChE activities. Also, the activity of COX-2 was reduced by 57.0 ± 4.4% under the conditions applied in this study. Additionally, all individual ultrafiltrates and PP exhibited anti-CAT, anti-SOD, anti-GPx, and anti-GR activities. This indicates the potential for positive modulation aimed at reducing oxidative stress through appropriate modifications of PP to enhance the activity of so-called “antioxidant” enzymes, without significantly compromising the most important activities of PP (i.e., anticholinesterase and anti-inflammatory effects). All tested fruits, as a rich source of anthocyanins, are beneficial for the treatment of AD, as highlighted in an excellent review by Suresh et al. [36]. However, there is a need to release the bioactive components in a programmed manner to ensure their delivery to the bloodstream in a controlled way. Therefore, we attempted to release bioactive compounds from our PP in a controlled manner. Hydrogels are convenient auxiliary materials for creating food gels. In previous studies, various gelling agents have been tested for their ability to slow down the release of bioactive compounds during digestion. Therefore, when selecting of papers to be used in the Discussion, we wanted to cite papers that meet the following criteria: (1) describe gelled edible structures (food products, dietary supplements or nutraceuticals) that entrap polyphenolic compound(s); (2) gelled products are based solely on gelatin or gelatin is the main (or at a least significant) gelling agent; (3) works regarding release characteristics of polyphenolic compounds during digestion (in vitro or in vivo). In our study, only pork gelatin was used as the gelling agent. Gómez-Mascaraque et al. [37] encapsulated (−)-epigallocatechin in a gelatin-chitosan hydrogel, achieving high encapsulation efficiency (95% ± 6%) and significantly delaying the degradation of the polyphenolic compound in aqueous solution (7-fold higher bioaccessibility of the polyphenol) after in vitro gastrointestinal digestion compared to a liquid solution. Peanparkdee et al. [38] encapsulated an extract from riceberry bran (with high TPC, TFvC, TAC, and antioxidant activity) using acid-treated gelatin (type A) and demonstrated that under simulated gastrointestinal conditions, gelatin capsules exhibited a lower degradation rate of antioxidants compared to the liquid extract. In another study, Peanparkdee et al. [39] embedded four bran extracts from Thai rice cultivars using gelatin, gum Arabic, and their mixture. In vitro digestion during gastric and intestinal phases revealed that microcapsules formed with gelatin exhibited the highest antioxidant activity. Encapsulation using gelatin and gum Arabic resulted in the lowest release of bioactive compounds and antioxidant activity after in vitro digestion. Dundar et al. [40] enriched “Boba balls”, composed of gelatin/sodium alginate, with pomegranate peel extract, reporting that during in vitro digestion, the extract was significantly preserved during the mouth and gastric phases, with the highest release of polyphenolic compounds (bioaccessibility) observed during the intestinal phase. Liu et al. [41] embedded anthocyanins in gelatin/gellan gums and noted retention of anthocyanins in gels during the “stomach” phase followed by subsequent release in the “intestine”. Silva et al. [42] embedded guaraná seed extracts in a gelatin/gum Arabic complex and reported that phenolic compound release was highest in simulated gastric fluid (reaching at least 80% cumulative release after 2 h). Martinović et al. [43] observed that sodium alginate alone or in combination with gelatin or chitosan effectively reduced the intestinal release of polyphenols (gallic acid, 3,4-dihydroxybenzoic acid, o-coumaric acid, epicatechin, and gallocatechin gallate) from phenol-rich grape pomace extract at rates ranging from 96.20% to 101.3%. Martinović et al. [44] entrapped grape pomace extract by ionic gelation of natural coatings (sodium alginate combined with maltodextrins, gelatin, chitosan, tragacanth, and Arabic gums), followed by air-, vacuum-, and freeze-drying of hydrogel microbeads. Among all products tested, alginate-based microbeads combined with gelatin, gum Arabic, and chitosan exhibited the most favorable in vitro release dynamics. Freeze-dried products were characterized by the highest cumulative release of polyphenols during the intestinal phase. Moreira et al. [45] conducted a randomized crossover trial comparing the consumption of a single dose of raw cinnamon (powder dissolved in water or in hard gelatin capsules). It was shown that entrapment of cinnamon in gelatin gel prolonged the release of bioactive compounds in patients. This resulted in a reduction in postprandial glucose spikes compared to patients who consumed cinnamon powder dissolved in water. Ozcan et al. [46] produced jelly candies fortified with purple basil leaf anthocyanin extract placed in gelatin and emulgel beads within gelatin. During digestion of both liquid extract and innovative jellies, authors observed reduced release of polyphenols during the “mouth” stage (8.27%) and “gastric” stage (74.44%) compared to free extract (24.92% and 86.13%, respectively). The release of polyphenols from emulgel beads placed in gelatin began during the “intestinal” stage, reaching 66.34–70.75% of the initial load; however, this modification increased the in vitro release of anthocyanins.

The limitations of our study should be pointed out. First, it must be strongly underlined that gelled products of other authors, presented in the Discussion, are different from each other (also from our product): they differ in composition, method of preparation, and/or additional modification of gelling agents (like gelatin in [38]). For the above reasons, it is difficult to make direct comparisons between individual publications and between them and our publication. Fortunately, a significant number of the cited works discussed the in vitro digestion process and the release of bioactives from the matrix. Importantly, these works convincingly point out two phenomena. A slowed release of polyphenolic compounds from the matrix, in comparison with the liquid form, during in vitro digestion, was observed. Also, the protection of the polyphenolic compound(s) against degradation as a result of encapsulation in the gel matrix. We confirmed the first phenomenon in our work, although we screened the subclasses of polyphenols. In the near future we plan to publish work in which we will indicate the changes in the concentration of individual polyphenolic compounds during in vitro digestion.

Another limitation of our work is the in vitro type of studies instead of in vivo experiments. However, the current work is the first one in the series and aimed to present, in great detail, the method of creating and studying PP and the food jelly as well as consumer perception in the form of a pilot study. The testing of PP using a rat model of AD is complete, and the publication of in vivo results is planned in the near future. Further stages of the research will be a study of consumer preferences for jelly beans on a large group of consumers (consumer market panel) and in vivo studies involving humans.

## 5. Conclusions

In this work, a polyphenol preparation was designed, developed, and comprehensively tested, and its presented features indicate the possibility of use in nutritional support for AD prevention. Due to its properties, it may also contribute to the overall improvement of brain health and cognitive functions, which is particularly important in the context of an aging society. The proposed food (gelatin-based jelly) can be used in people of all ages, because it is a food with a natural composition. The results suggest that this new composition, combined with the proposed delivery method, has great potential to be used as a functional ingredient in products for oral administration, aimed at controlling the release of bioactive compounds in the gastrointestinal tract.

A limitation in the presented work is the use of biochemical in vitro methods to determine biological activities, as well as mimicking the absorption of biologically active compounds by in vitro digestion. However, in the near future, the results regarding the effect of this polyphenol preparation on cognitive parameters in a rat model with induced AD will be presented.

## Figures and Tables

**Table 1:** Composition of individual ultrafiltrates and PP (in fresh mass).

Concentrated Ultrafiltrate From:	TPC (mg GAE/g)	TFvC (mg Quercetin/g)	TFnC (mg Catechin/g)	TCT (mg Catechin/g)	TAC (µg cya-3-glu/g)
Apricot	6.11 ± 0.32	3.22 ± 0.01	0.03 ± 0.01	1.45 ± 0.08	3.07 ± 0.25
Blueberry	12.08 ± 0.41	3.56 ± 0.02	1.08 ± 0.01	4.62 ± 0.09	429.33 ± 1.03
Chokeberry	17.58 ± 0.48	7.00 ± 0.02	3.08 ± 0.01	4.94 ± 0.38	673.95 ± 1.98
Cranberry	13.11 ± 0.35	6.39 ± 0.03	2.04 ± 0.00	2.55 ± 0.16	42.35 ± 0.82
Peach	3.96 ± 0.11	1.34 ± 0.03	0.32 ± 0.01	1.23 ± 0.09	5.70 ± 0.36
Raspberry	8.79 ± 0.34	3.44 ± 0.01	0.27 ± 0.01	3.10 ± 0.10	158.32 ± 1.83
W. strawberry	12.57 ± 0.46	7.14 ± 0.04	0.65 ± 0.01	1.69 ± 0.32	139.32 ± 2.83
PP	8.10 ± 0.45	1.57 ± 0.03	0.42 ± 0.01	3.54 ± 0.22	53.16 ± 0.40

**Table 2:** Retention times of authentic HPLC standards and concentrations detected in PP.

Compound	Retention Time (min)	Concentration (μg/g)
3,4,5-Trihydroxybenzoic acid (Gallic acid)	7.90	531.8 ± 10.5
3,4-di-OH-Benzoic acid (Protocatechuic acid)	25.57	85.0 ± 3.6
2,5-di-OH-Benzoic acid (Gentisic acid)	26.81	255.5 ± 10.0
3-Cafeorylquinic acid (Chlorogenic acid)	34.47	446.8 ± 29.1
4-OH-3-OCH_3_-Benzoic acid (Vanillic acid)	36.04	96.4 ± 4.1
(−)-Epicatechin	37.04	134.5 ± 5.9
4-OH-Benzoic acid	37.61	49.1 ± 3.2
3,4-di-OH-Cinnamic acid (Caffeic acid)	39.66	229.5 ± 15.5
4-OH-3,5-di-OCH_3_-Benzoic acid	42.00	116.8 ± 7.7
4-OH-Cinamic acid (***p***-Coumaric acid)	42.71	48.2 ± 2.7
4-OH-3-OCH_3_-Cinnamic acid (Ferulic acid)	43.62	21.4 ± 1.4
Cyjanidin-3-glucoside	44.11	472.7 ± 6.4
Ellagic acid	46.50	2694.1 ± 20.9
Pelargonidin 3-*O*-glucoside	47.07	253.6 ± 15.0
Phloretin 2′-β-D-glucoside (Phloridzin)	48.30	16.4 ± 1.6
2-OH-Benzoic acid (Salicylic acid)	50.30	217.3 ± 6.8
Quercetin 3-rutinoside	51.00	150.9 ± 0.9
Quercetin 3-glucoside	52.30	136.4 ± 3.2
Quercetin	53.15	167.3 ± 0.5
Delphinidin	54.91	209.1 ± 10.9
Pelargonidin	63.26	408.2 ± 27.3
Kaempferol 3-rutinoside	66.13	42.7 ± 2.3
Cinnamic acid	67.43	4.5 ± 0.5
Procyanidin B2	70.60	581.4 ± 1.4
Cyanidin	72.20	60.5 ± 0.9
Kaempferol	74.12	20.5 ± 0.5

**Table 3:** Effect of individual ultrafiltrates and PP on enzymes’ activities.

Concentrated Ultrafiltrate from:	Inhibition of AChE	Inhibition of BChE	Inhibition of Catalase	Inhibition of SOD	Inhibition of GPx	Inhibition of GR
	(%)				(nmol Consumed NADPH/Min mg Sample)	
Apricot	40.8 ± 0.9	28.5 ± 1.0	30.1 ± 18.1	73.5 ± 8.9	0.36 ± 0.06	2.22 ± 0.32
Blueberry	46.8 ± 0.1	43.1 ± 0.9	28.8 ± 8.8	74.7 ± 9.9	0.21 ± 0.03	7.39 ± 0.03
Chokeberry	35.4 ± 1.2	53.8 ± 1.2	100.0 ± 8.6	93.6 ± 4.1	0.87 ± 0.10	5.54 ± 0.13
Cranberry	23.1 ± 2.2	31.5 ± 0.6	62.1 ± 37.8	61.9 ± 6.1	1.85 ± 0.05	7.99 ± 0.22
Peach	80.3 ± 0.2	62.5 ± 0.2	71.5 ± 12.0	82.4 ± 5.0	0.69 ± 0.11	2.54 ± 0.12
Raspberry	62.5 ± 0.2	46.9 ± 1.5	22.4 ± 18.5	65.8 ± 1.3	1.87 ± 0.08	2.17 ± 0.15
Wild Strawberry	74.1 ± 0.0	73.1 ± 0.4	94.8 ± 7.8	70.8 ± 6.8	1.26 ± 0.04	6.69 ± 0.01
All combined (PP)	55.7 ± 0.3	38.5 ± 1.7	66.2 ± 6.4	76.7 ± 3.3	0.63 ± 0.08	3.37 ± 0.19

**Table 4:** Antioxidant activity parameters of individual ultrafiltrates and PP.

Concentrated Ultrafiltrate	FRAP TEAC (μg Troloxu/g)	CUPRAC (mmol Quercetin/L)	HORAC (GAE, µg Gallic Acid/mL)	ABTS	DPPH
				(TEAC μg Troloxu/mL)	(TEAC μg Troloxu/mL)
Apricot	1.78 ± 0.01	0.07 ± 0.09	5.2 ± 0.9	8.08 ± 0.27	0.12 ± 0.01
Blueberry	6.42 ± 0.05	0.71 ± 0.04	8.1 ± 0.1	1.48 ± 0.10	0.05 ± 0.00
Chokeberry	20.05 ± 0.03	1.93 ± 0.03	8.5 ± 0.8	6.11 ± 0.03	0.04 ± 0.01
Cranberry	11.96 ± 0.10	0.83 ± 0.06	39.1 ± 0.0	4.49 ± 0.00	0.06 ± 0.01
Peach	1.67 ± 0.10	0.09 ± 0.01	5.7 ± 0.3	7.81 ± 0.54	0.14 ± 0.02
Raspberry	4.38 ± 0.03	0.37 ± 0.07	34.0 ± 0.2	1.74 ± 0.16	0.06 ± 0.01
Wild Strawberry	9.04 ± 0.01	0.89 ± 0.01	16.0 ± 0.1	4.06 ± 0.01	0.06 ± 0.01
All combined (PP)	6.25 ± 0.04	0.27 ± 0.01	5.5 ± 0.2	4.50 ± 0.06	0.08 ± 0.01

## Data Availability

Data is contained within the article and Supplementary Materials.

## Supplementary Materials

The following supporting information can be downloaded at: https://www.mdpi.com/article/10.3390/antiox14050535/s1, Table S1: Dry mass of individual ultrafiltrates and PP; Table S2: Retention times of authentic HPLC standards and fragmentation parameters of the main polyphenolic compounds detected in PP; Table S3: Quantitative analysis of selected compounds present in PP; Table S4: Gelling agents used; Table S5: Significant disintegration of jellies during in vitro Digestion; Table S6: Dry mass of samples during in vitro Digestion; Figure S1: Chromatograms of HPLC standards; Figure S2: Apricot—compounds present in the ultrafiltrate (5 kDa), analytical HPLC-UV at 280 nm; Figure S3: Peach—compounds present in the ultrafiltrate (5 kDa), analytical HPLC-UV at 280 nm; Figure S4: Phenolic compounds from peach separated by polyvinylpolypyrrolidone (PVPP), analytical HPLC, 280 nm; Figure S5: Example of identification of a phenolic compound (cyanidin-3-rutinoside) from peach extract. Cyanidin-3-rutinoside was purified by adsorption on PVPP followed by analytical HPLC chromatography; Figure S6: Free phenolic acid present in peach ultrafiltrate (5 kDa), 280 nm (two independent extractions); Figure S7 shows an example of a chromatogram of the final PP; Figure S7: PP chromatogram (analytical HPLC, UV detection at 280 nm (blue), 365 nm (green), 520 nm (red)); Figure S8: Example MS/MS spectra of compounds present in PP; Figure S9: Prototype jellies produced using gelatin; Figure S10: In vitro digestion model used in this study.
