# Supplementary material for: Encapsulation of Polyphenolic Preparation in Gelatin Fruit Jellies Slows the Digestive Release of Cholinesterase Inhibitors In Vitro
Source: Antioxidants (Basel). 2025 Apr 29;14(5):535. doi: 10.3390/antiox14050535 (PMC12108259; doi:10.3390/antiox14050535)
Supplement: Supplementary file 1 [file antioxidants-14-00535-s001.zip › antioxidants-3566518-supplementary.pdf]

Supplementary Materials

Table S1. Dry mass of individual ultrafiltrates and PP.

Sample	Dry mass (%)
Concentrated peach ultrafiltrate	71.4 ± 2.6
Concentrated raspberry ultrafiltrate	70.2 ± 0.1
Concentrated blueberry ultrafiltrate	67.2 ± 0.1
Concentrated cranberry ultrafiltrate	66.7 ± 0.3
Concentrated wild strawberry ultrafiltrate	66.2 ± 0.4
Concentrated apricot ultrafiltrate	64.7 ± 1.3
Concentrated chokeberry ultrafiltrate	76.1 ± 2.0
PP	67.5 ± 0.6

Analytical LC with UV, spectrofluorimetric, and MS detection

Compounds present in the ultrafiltrates from individual fruits as well as in the PP were identified based on the retention times of authentic chromatographic standards of phenolic compounds (Sigma-Aldrich, Fluka, USA; Extrasynthese, France; Roth, Germany; Apin Chemicals, UK) (Figure S1).

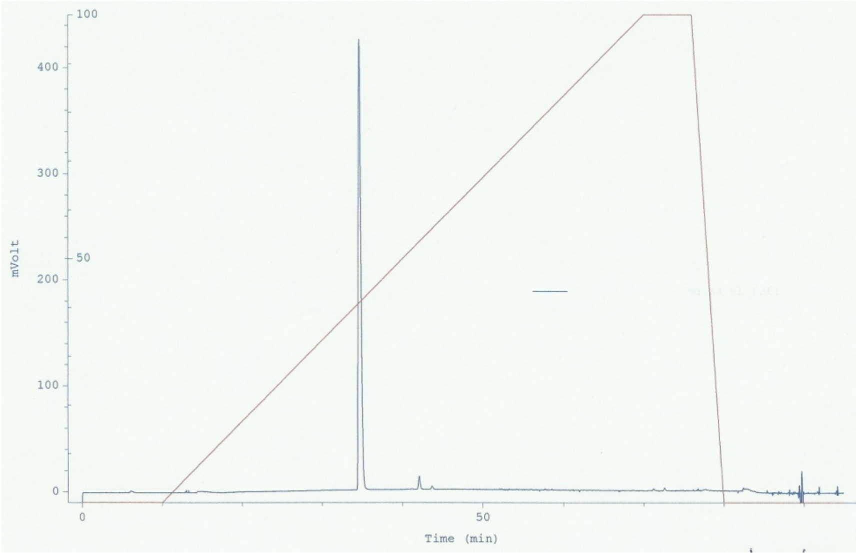

3-OH-Benzoic acid

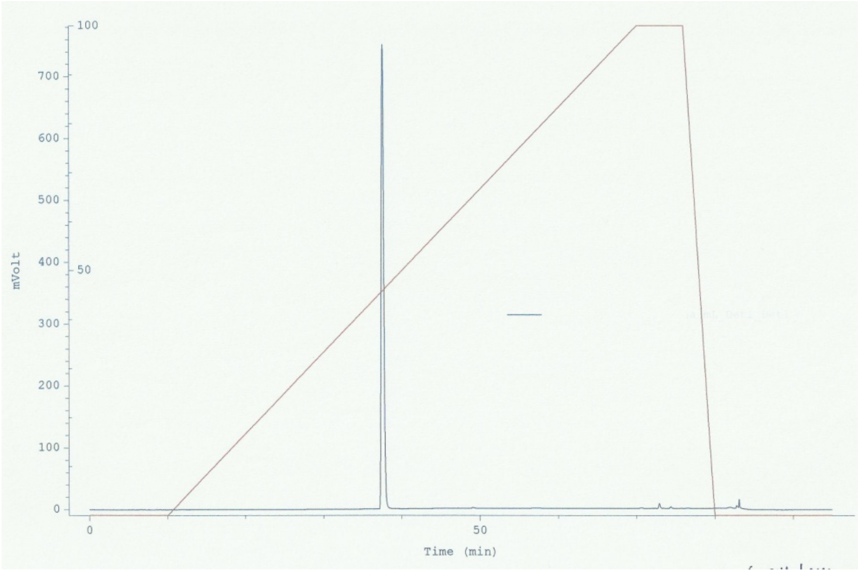

4-OH-Benzoic acid

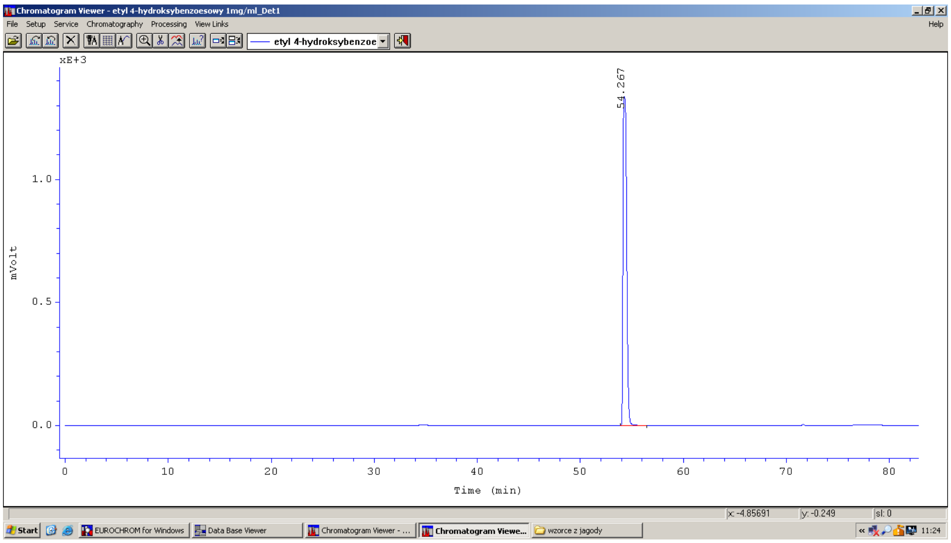

Ethyl 4-OH-benzoate

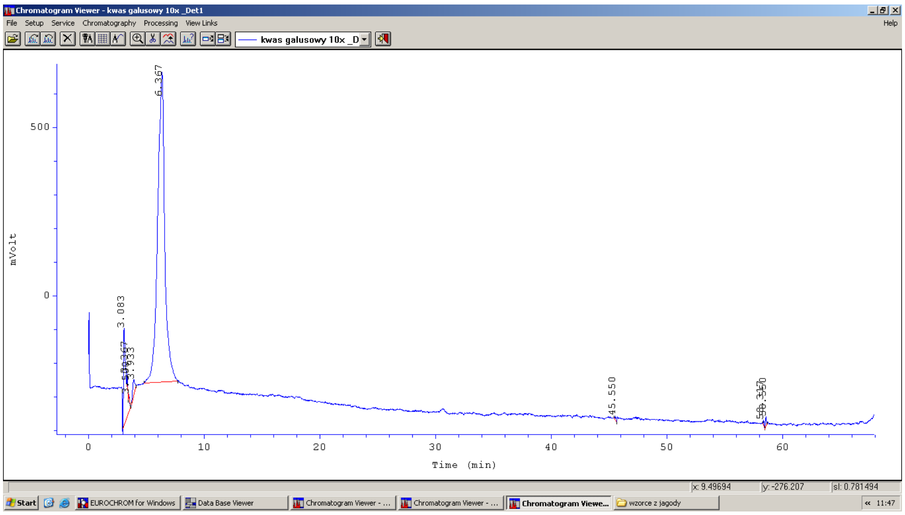

Gallic acid

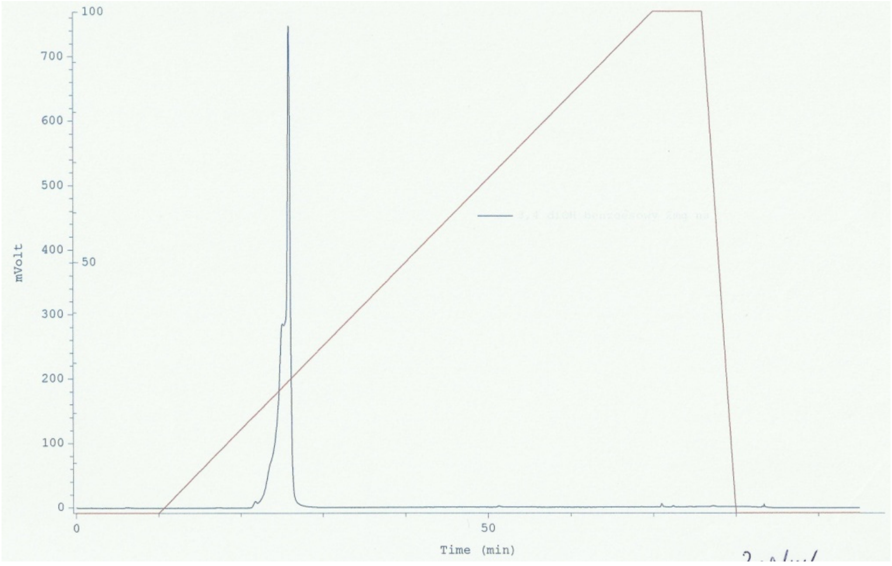

3,4- Di-OH-benzoic acid

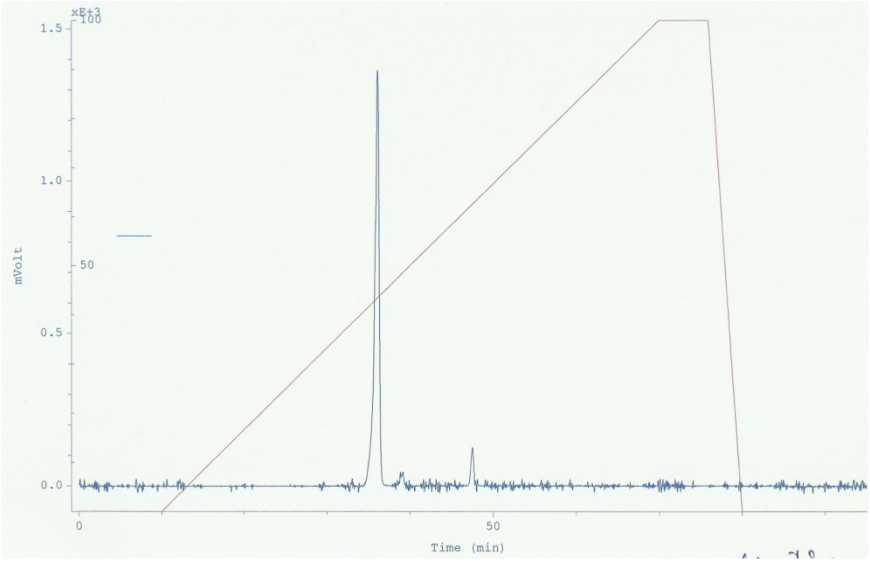

Vanillic acid

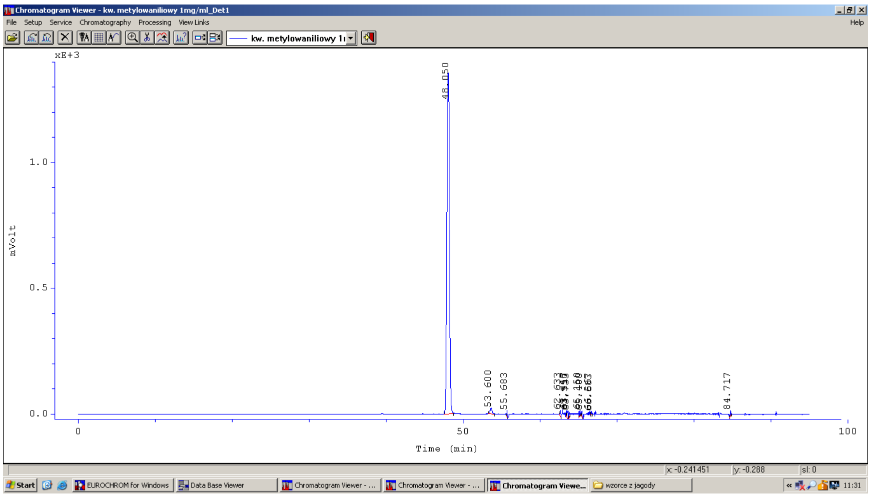

Methyl vanillate

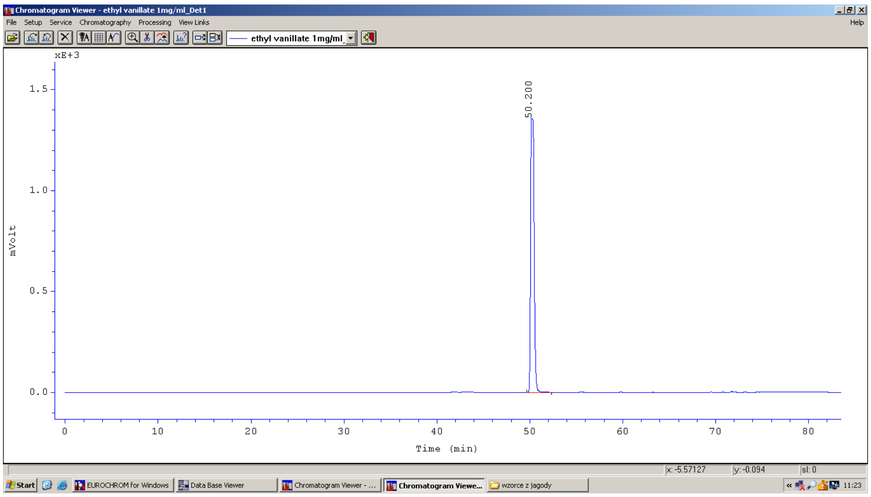

Ethyl vanillate

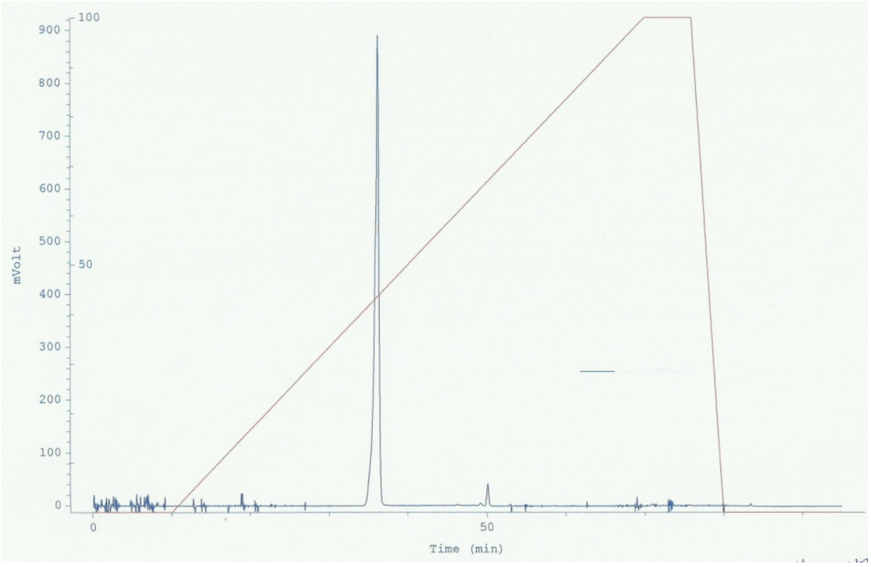

Homovanillic acid

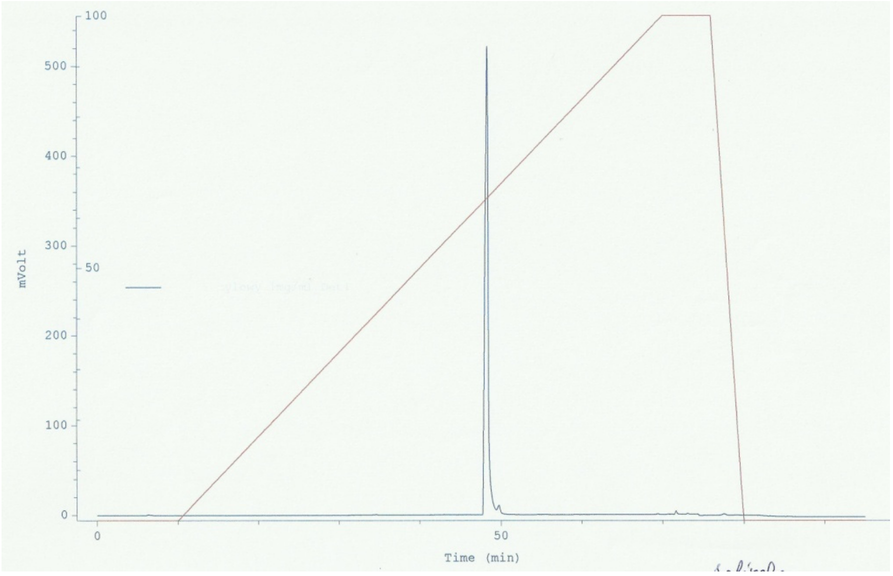

Salicylic acid

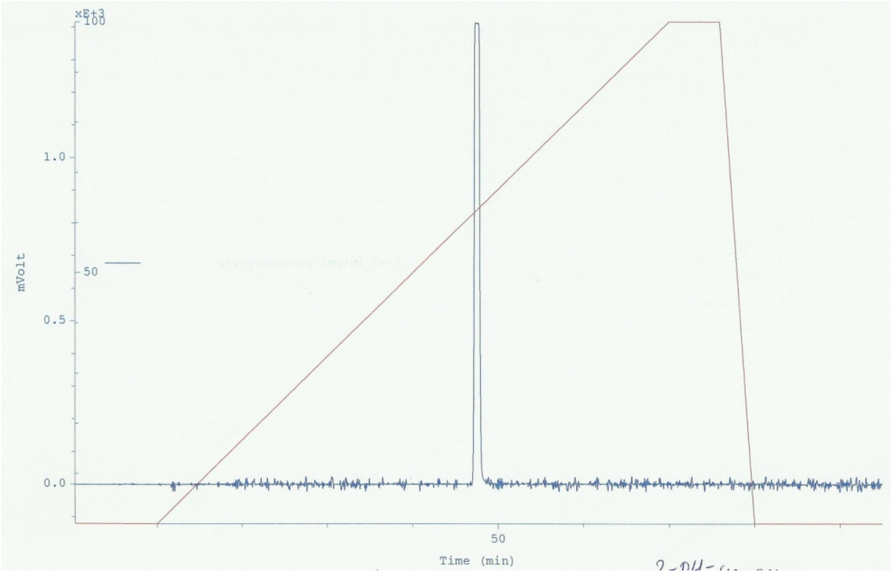

2-Coumaric acid

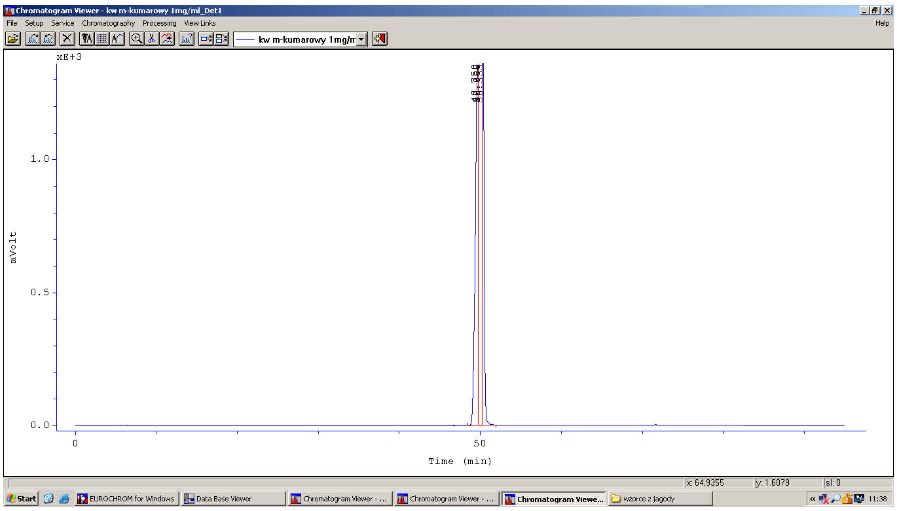

3-Coumaric acid

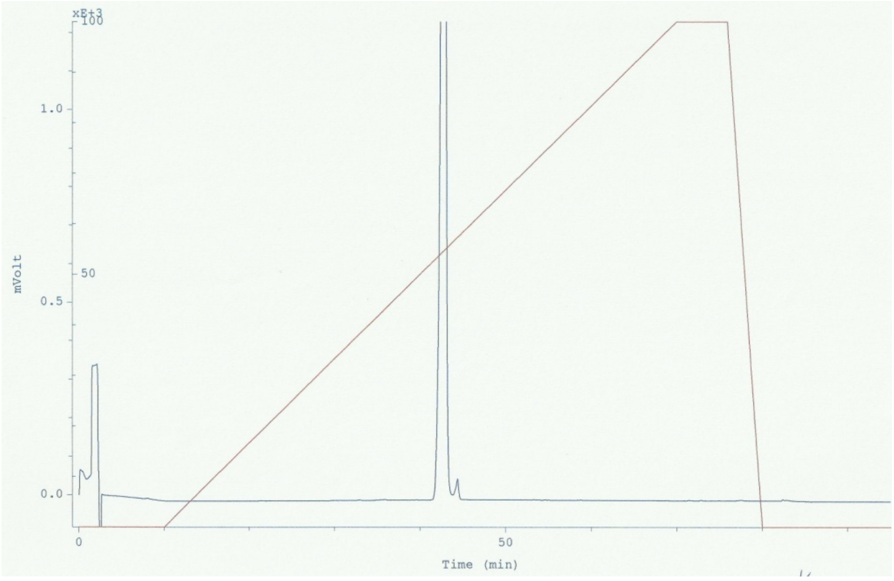

4-Coumaric acid

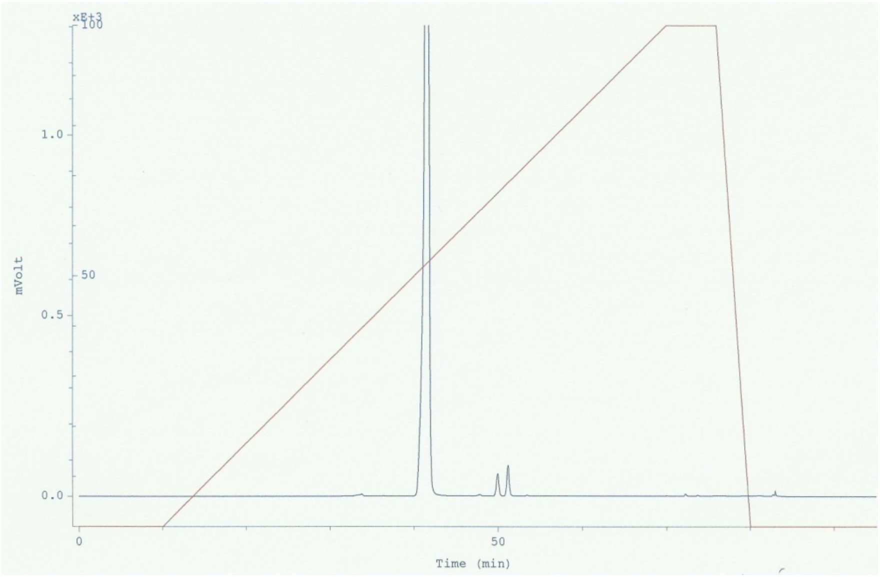

Syringic acid

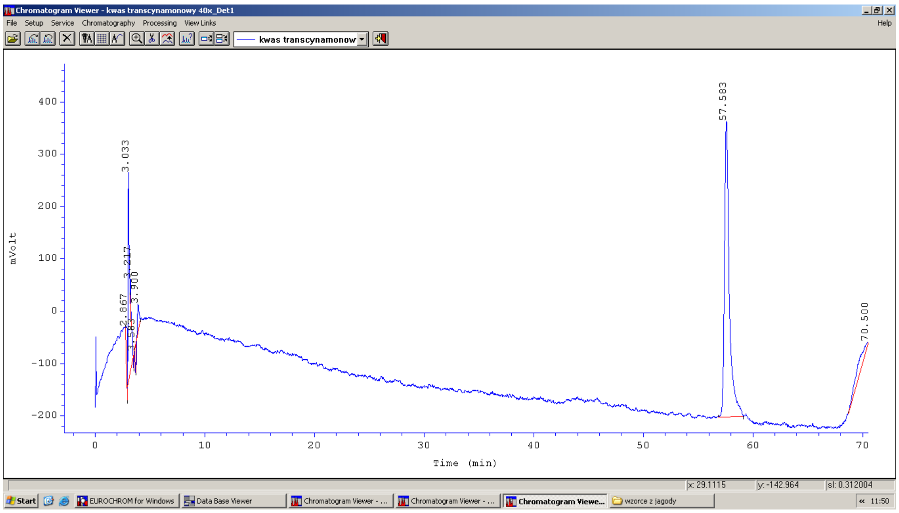

trans-Cinnamic acid

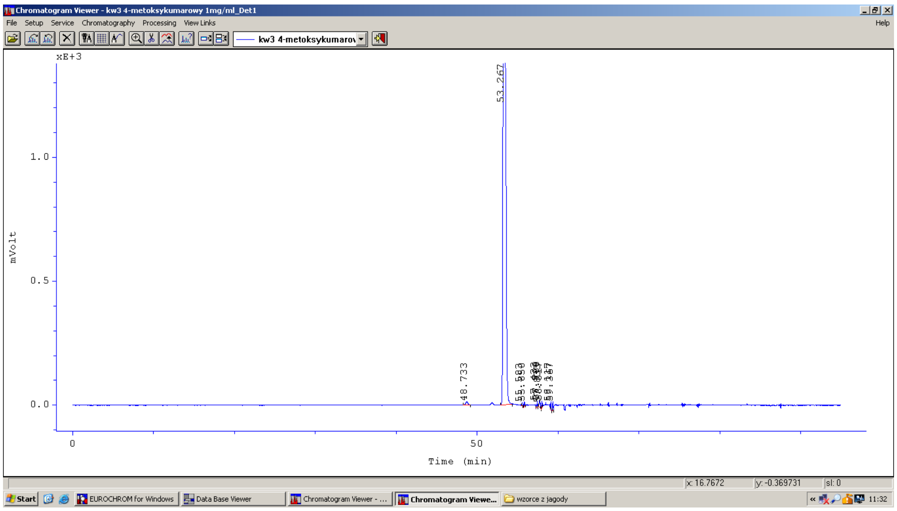

Methyl 4-coumarate

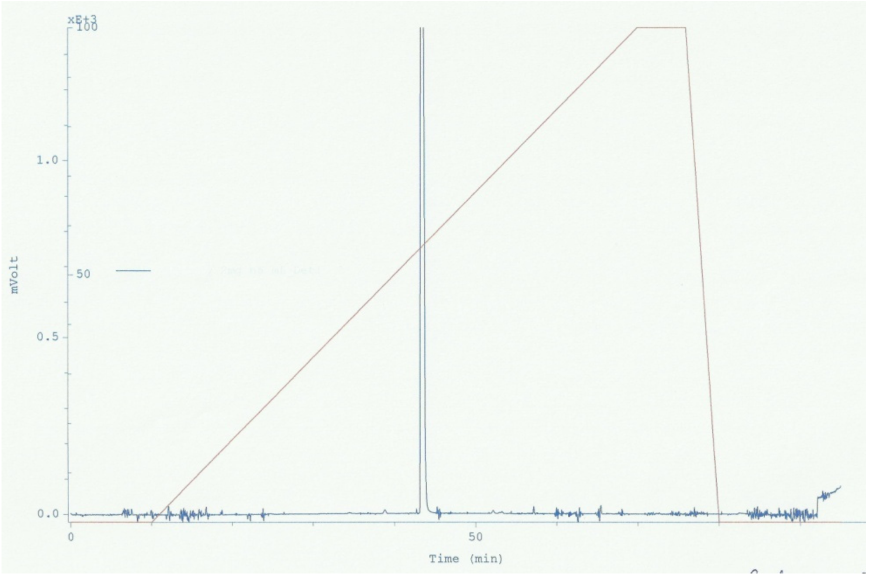

Ferulic acid

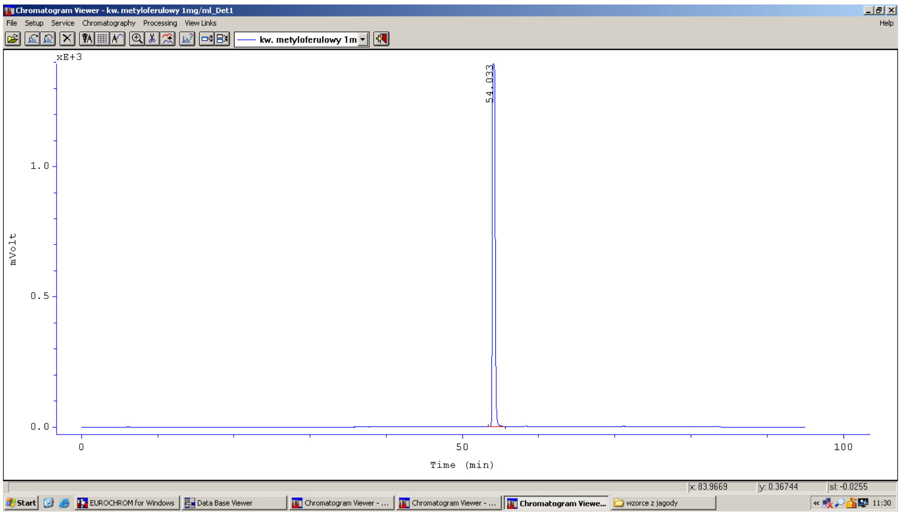

Methyl ferulate

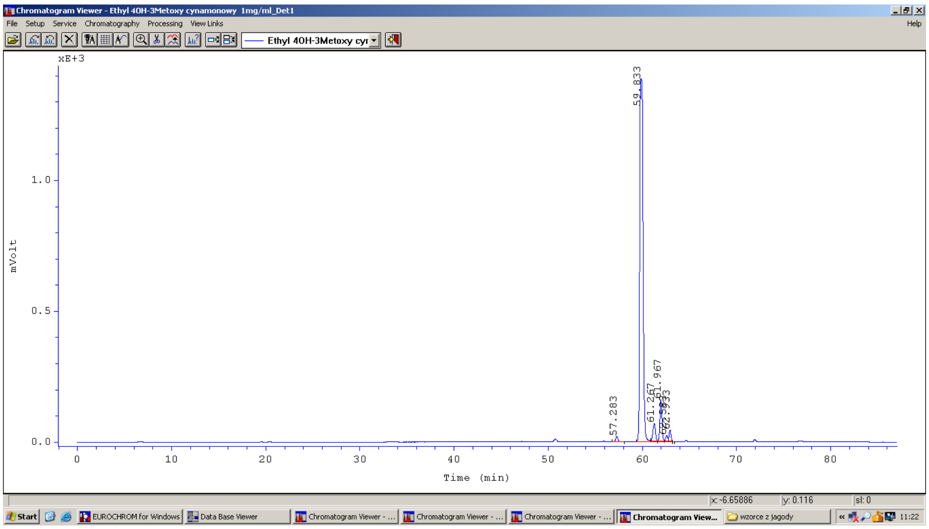

Ethyl ferulate

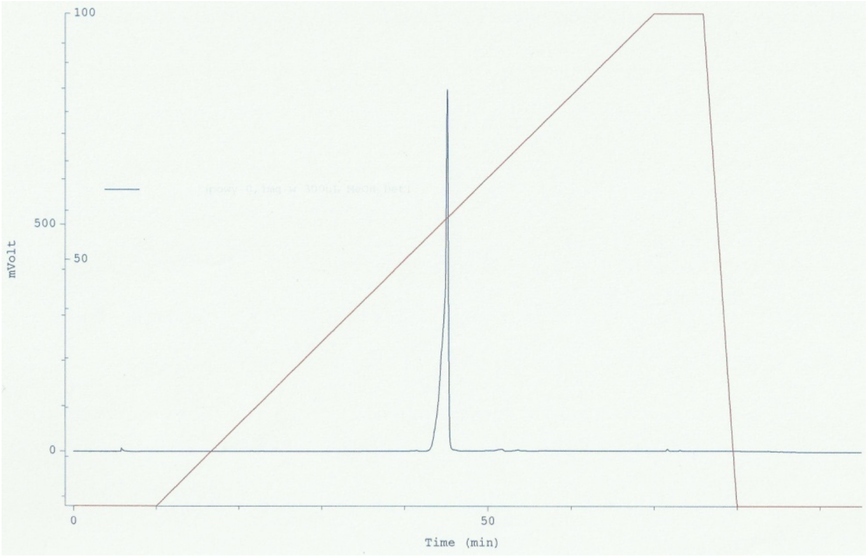

Sinapic acid

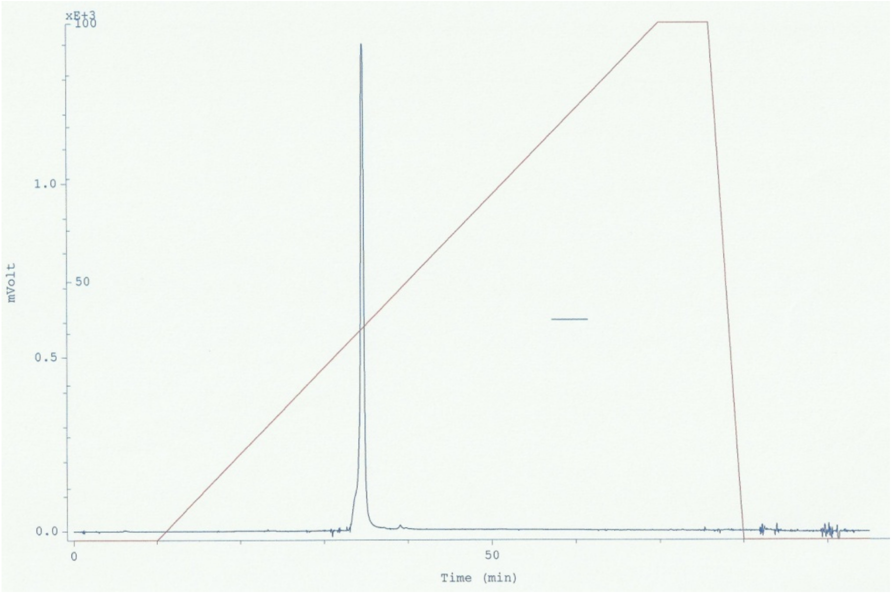

Chlorogenic acid

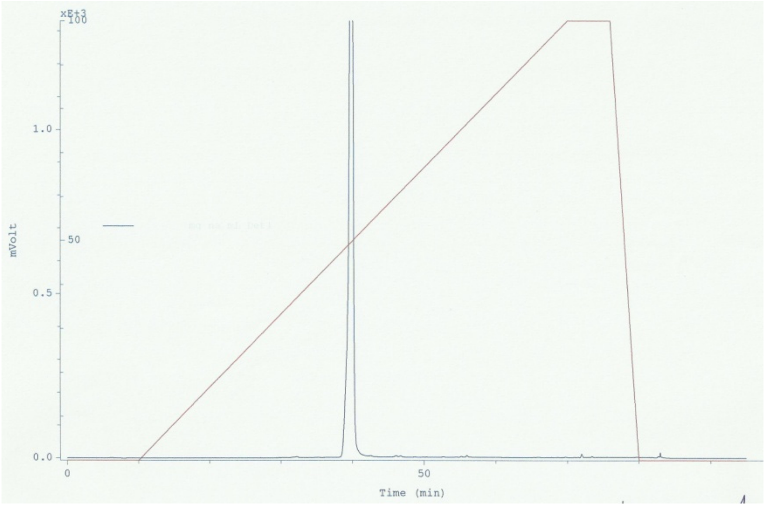

Caffeic acid

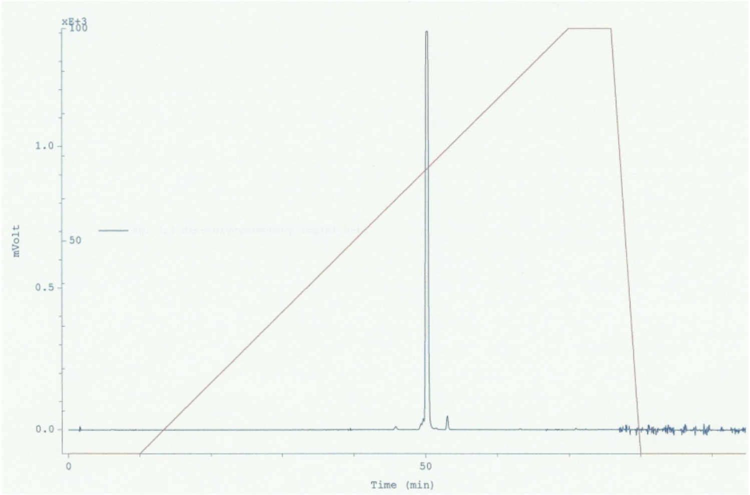

Dimethyl caffeic acid ether

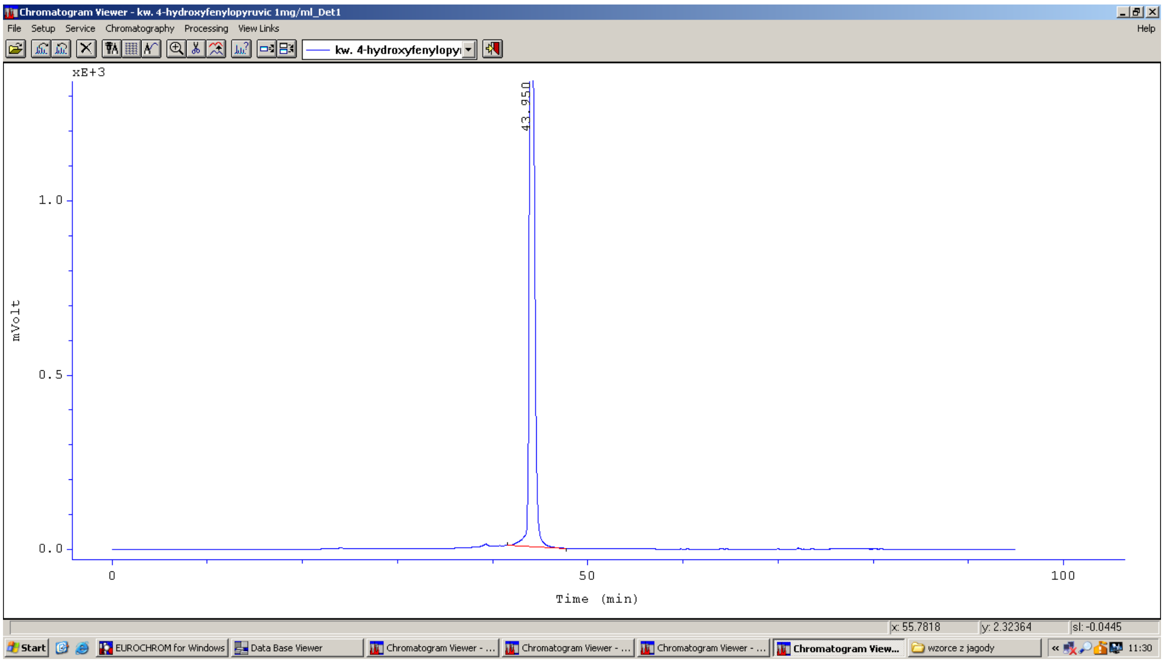

4-OH-Phenylpyruvic acid

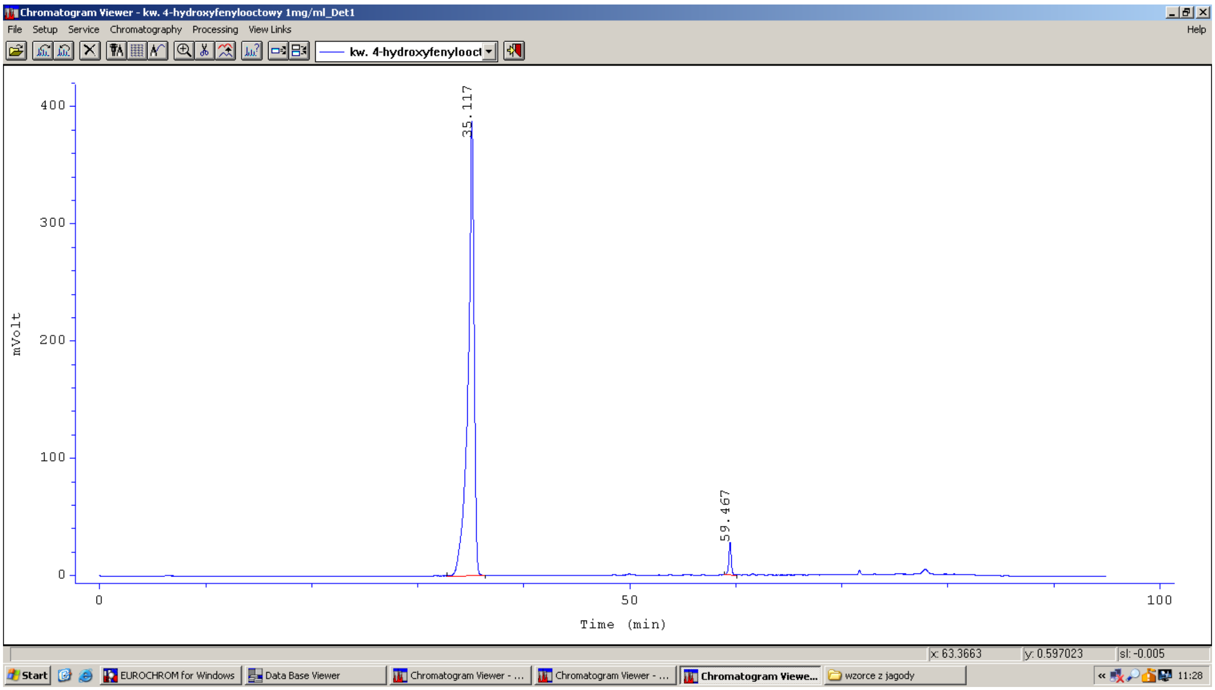

4-OH-Phenylacetic acid

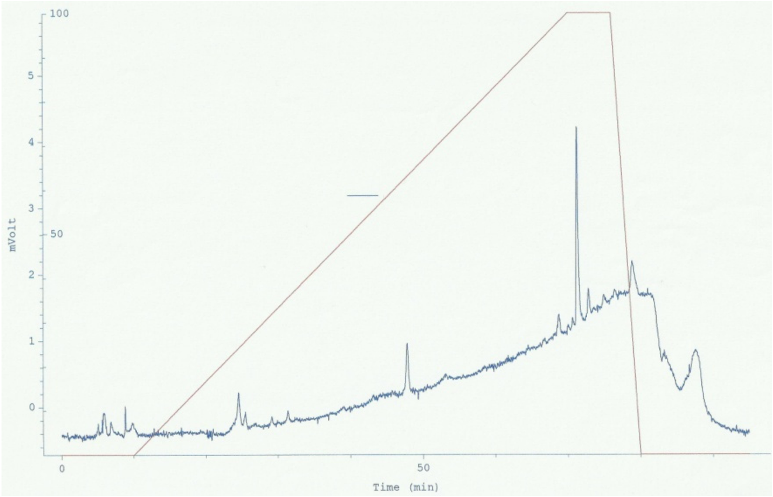

Cyanidin

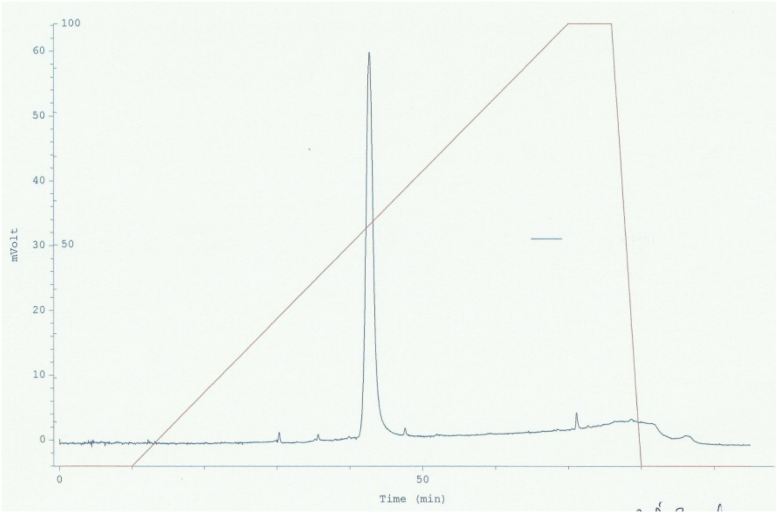

Cyanidin 3-glucoside

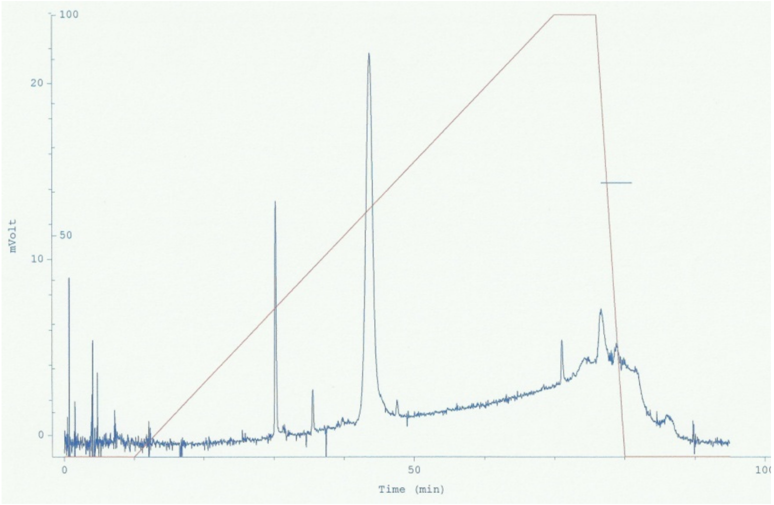

Cyanidyno 3-rutinoside

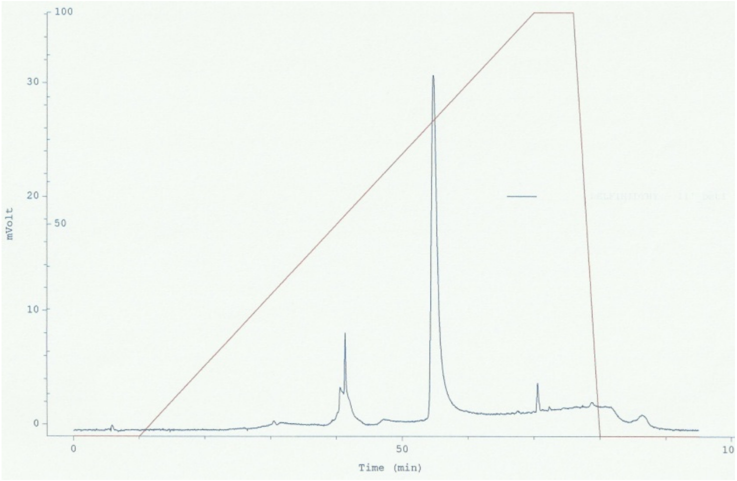

Delphinidin

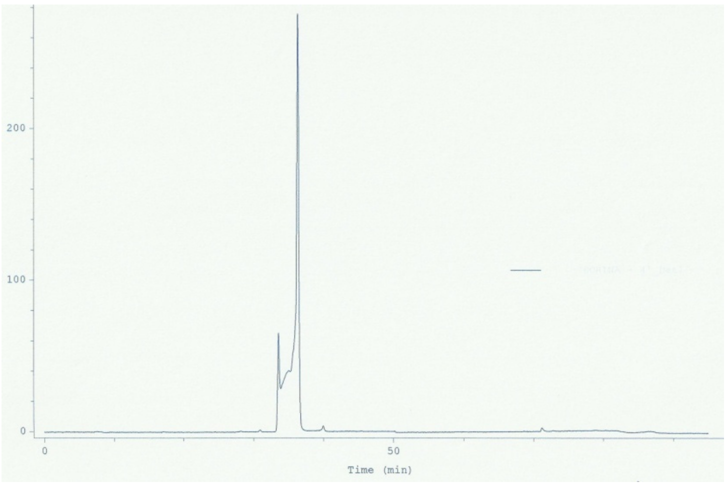

Epicatechin

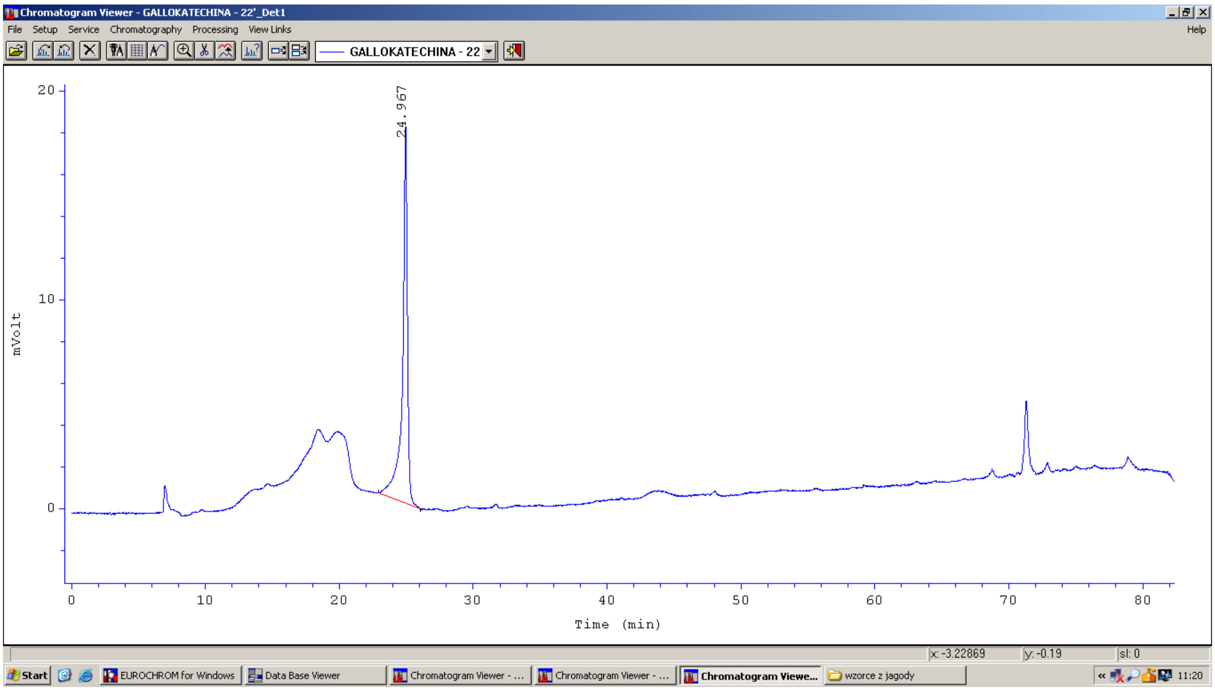

Gallocatechin

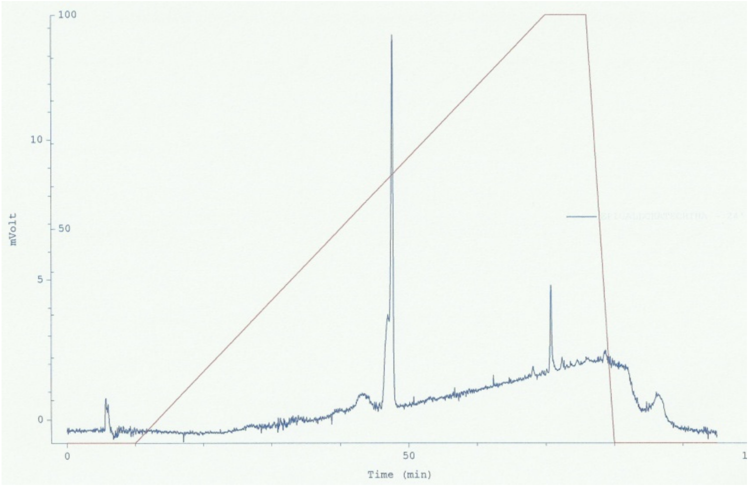

Epigallocatechin

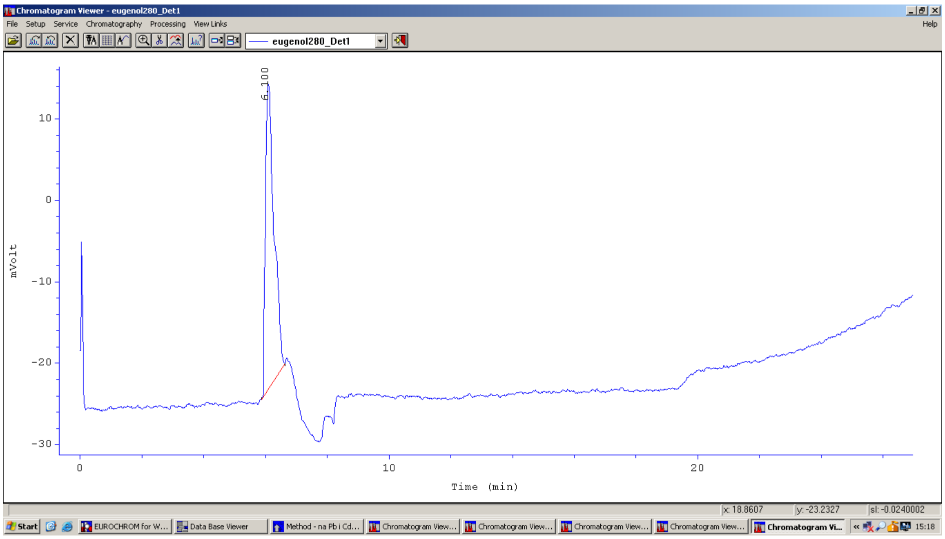

Eugenol

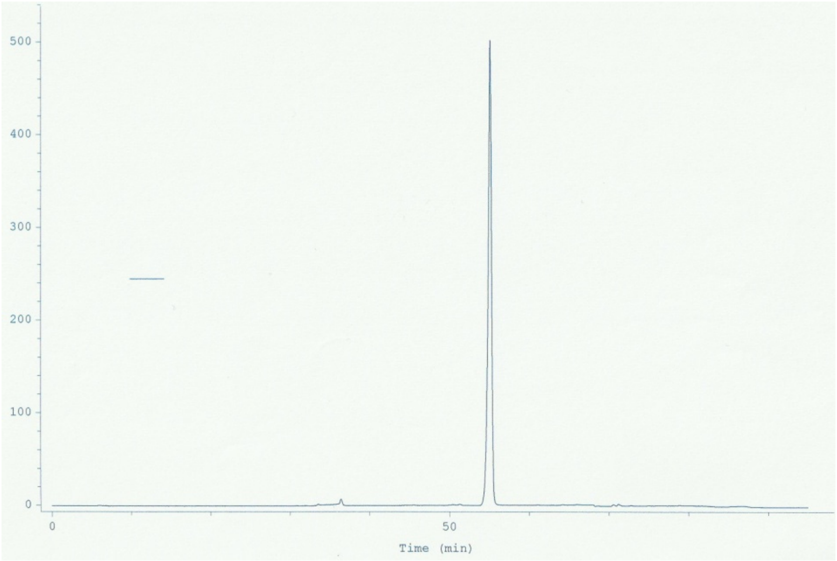

Phloretin

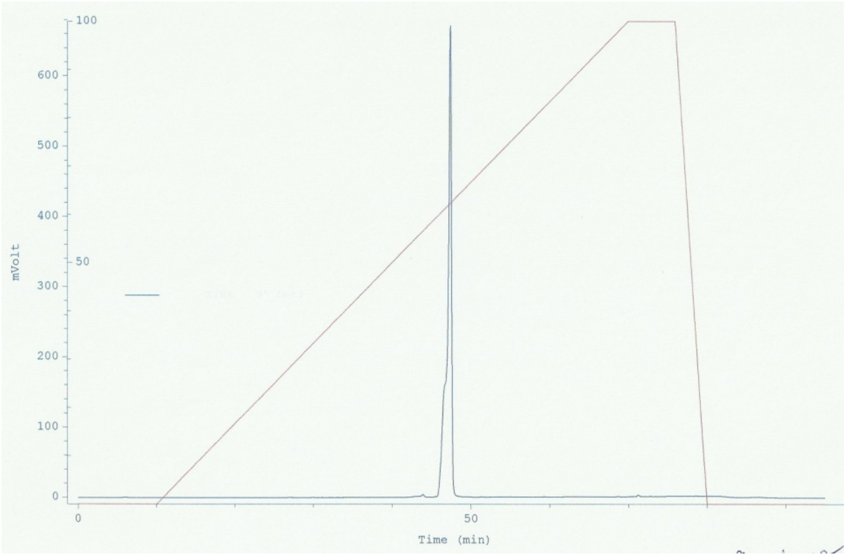

Phloridzin

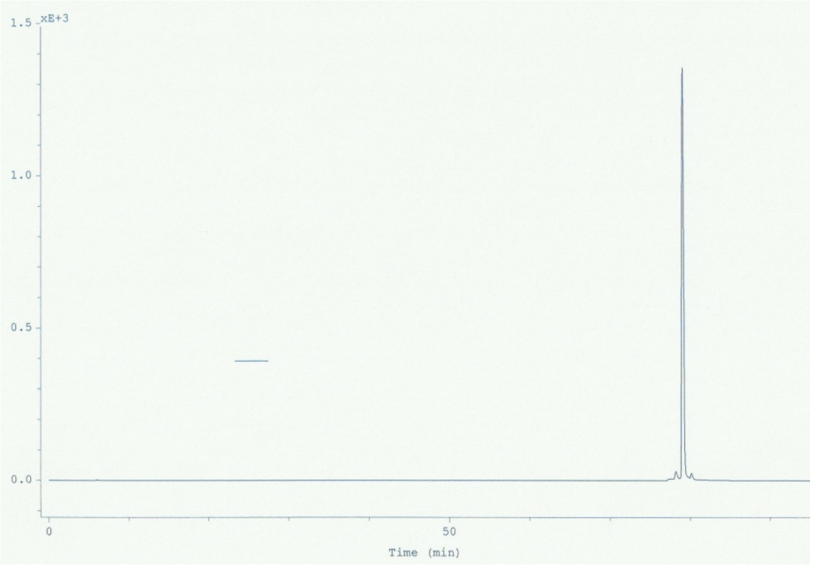

Hesperetin

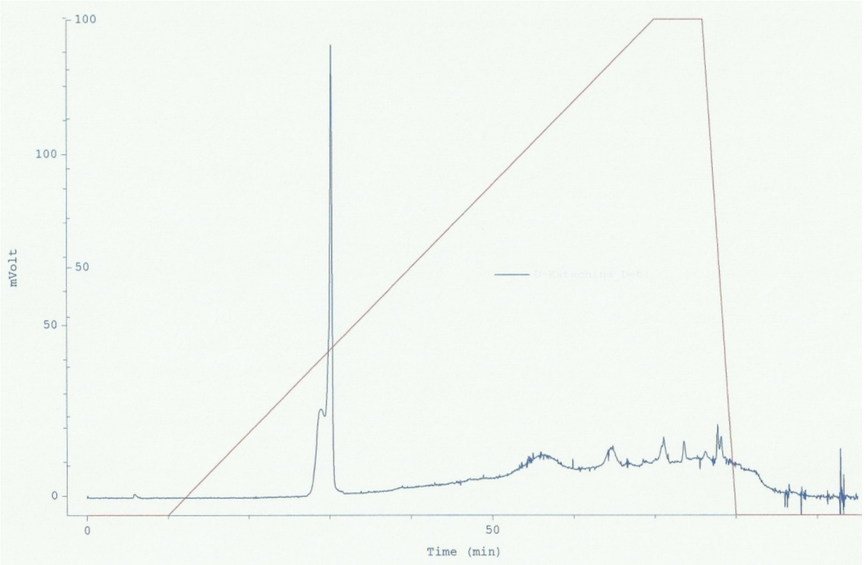

(+)-Catechin

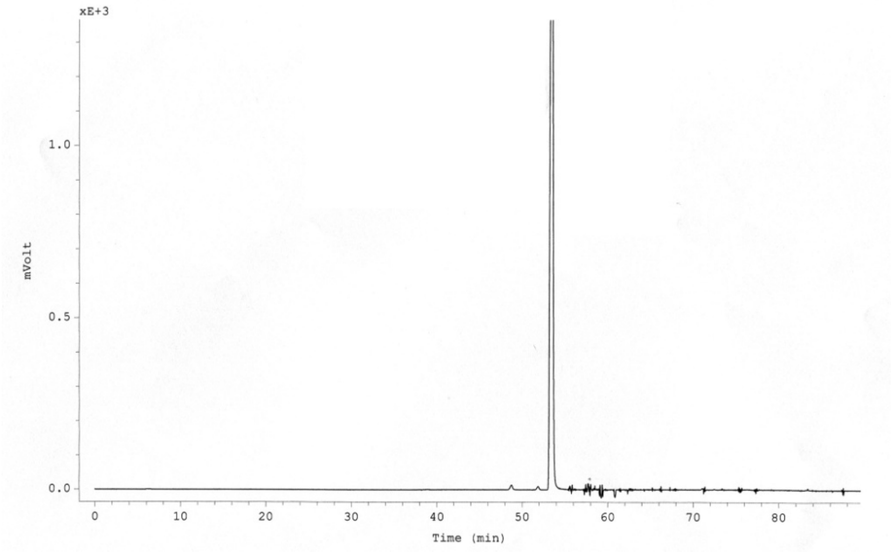

Quercetin

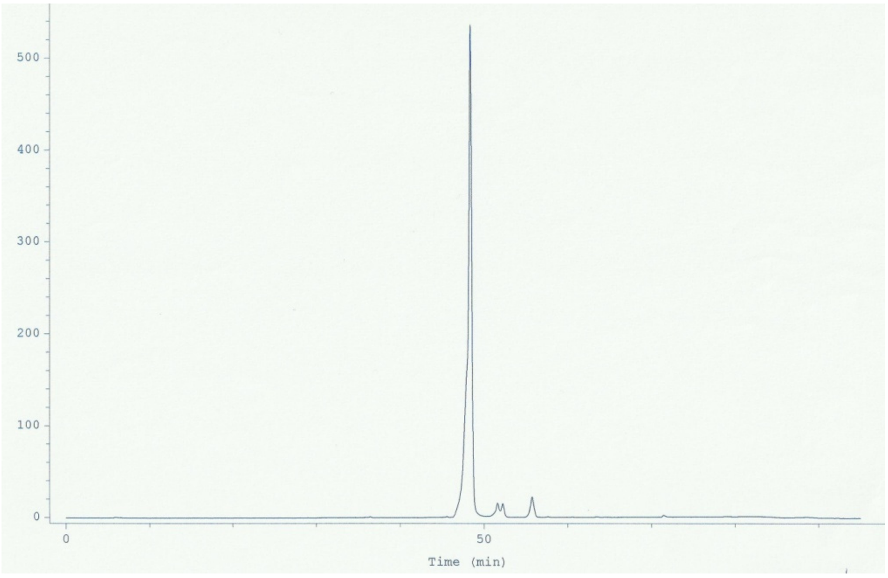

Rutin

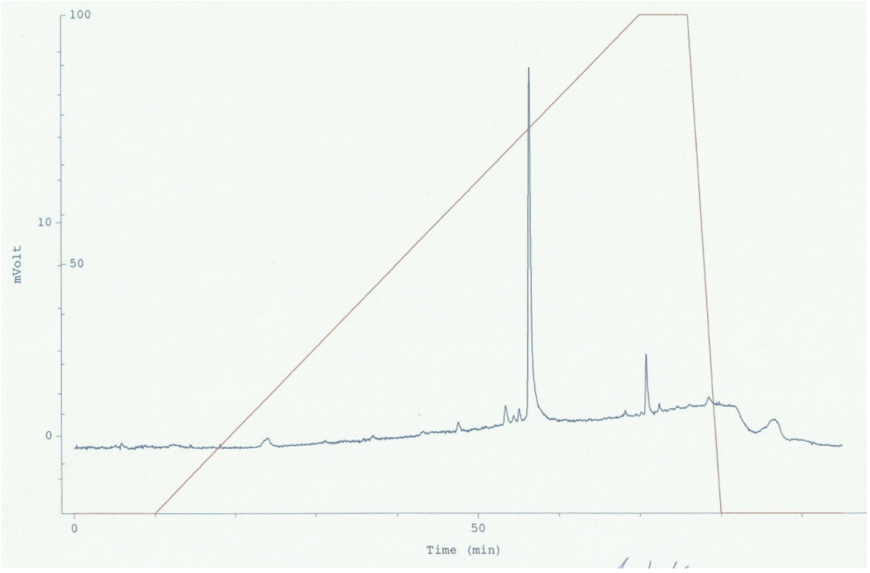

Luteolin

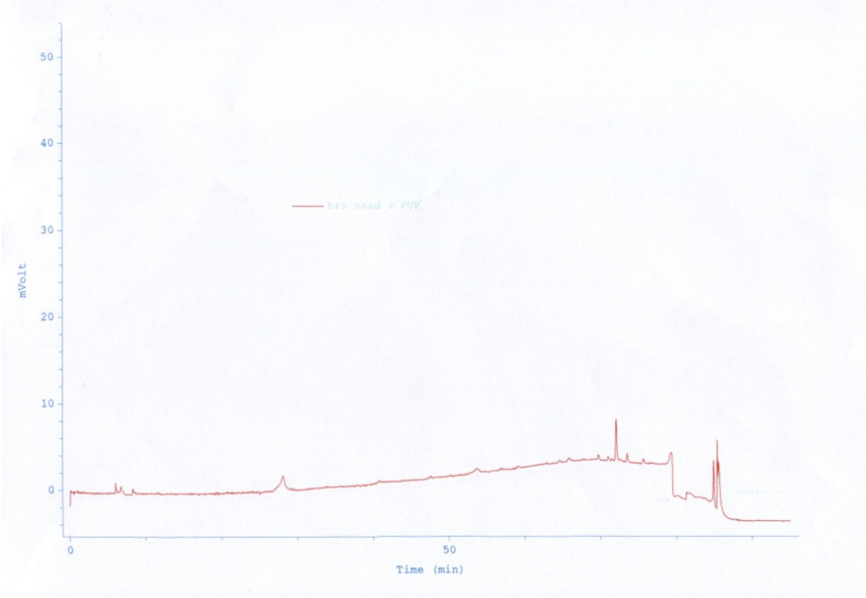

Apigenin

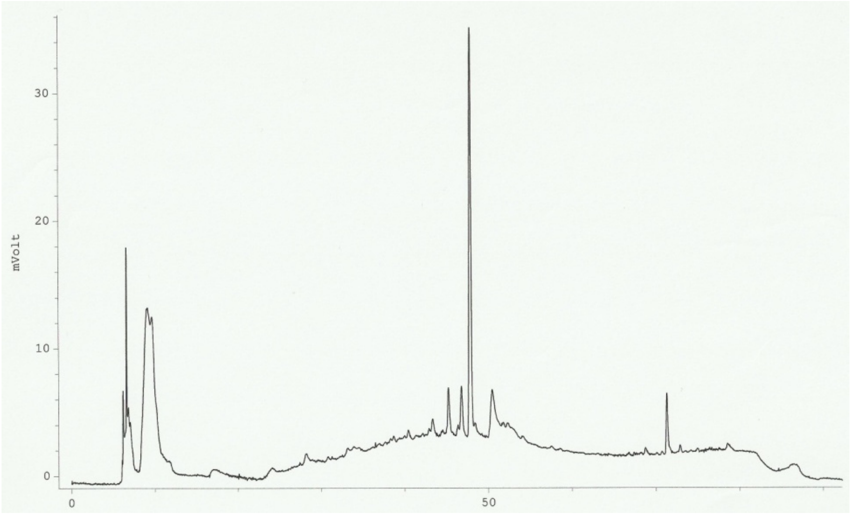

Myricetin

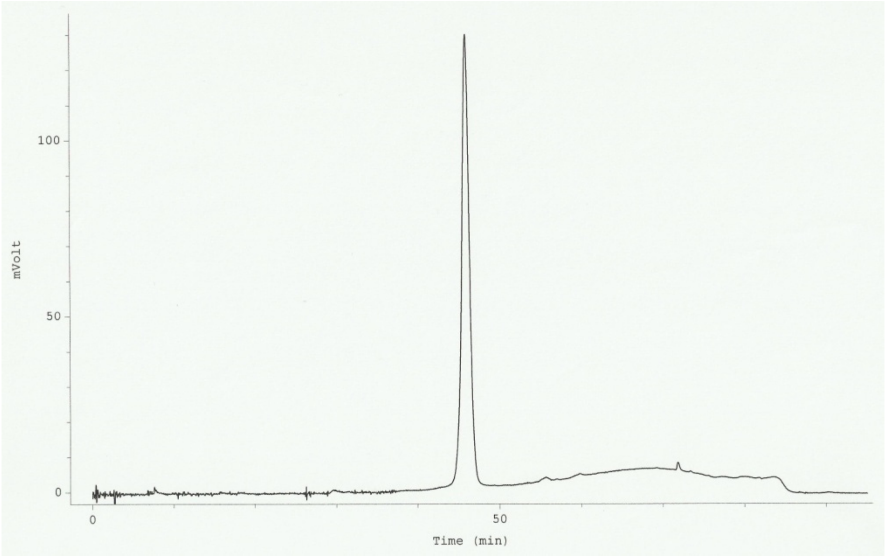

Pelargonidin 3-O-glucoside

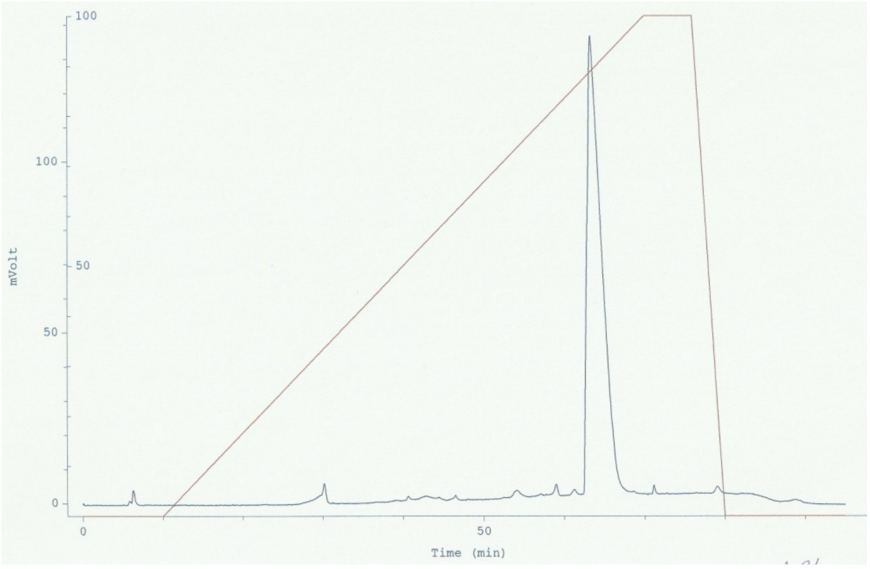

Pelargonidin

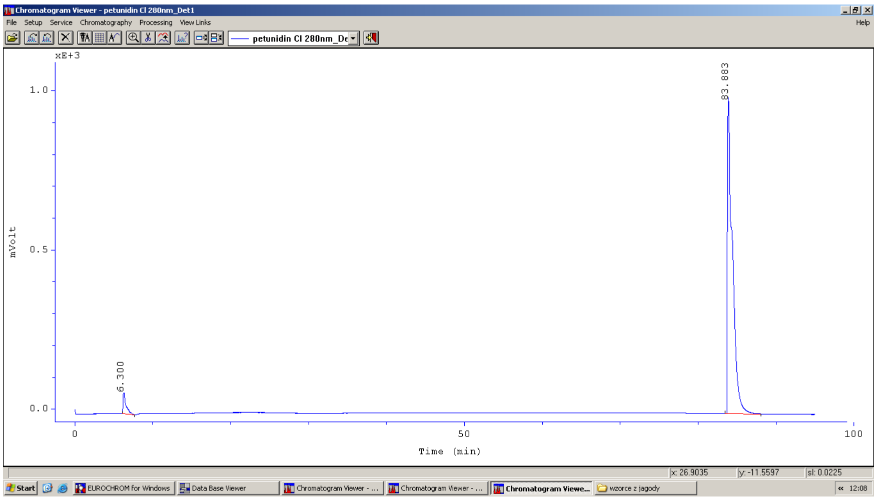

Petunidin

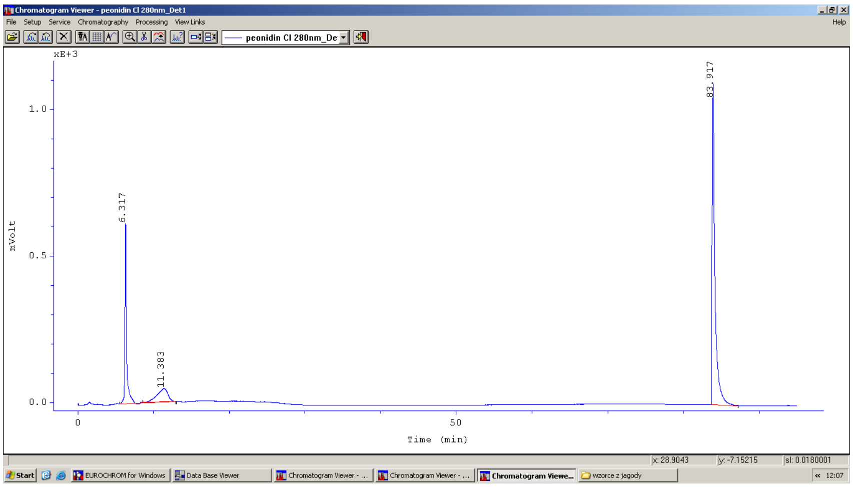

Peonidin

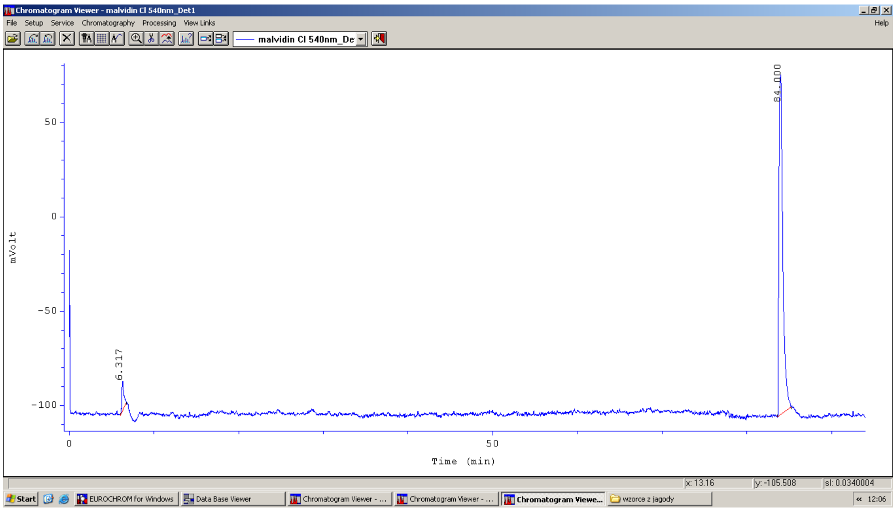

Malvidin

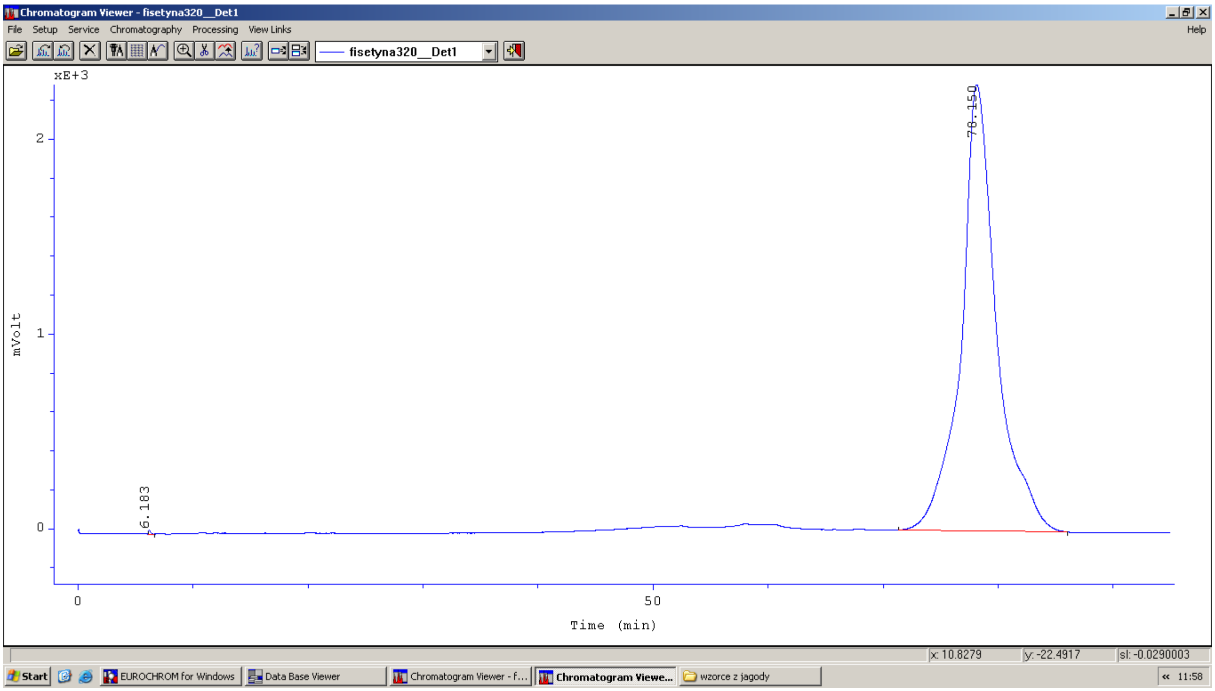

Fisetin

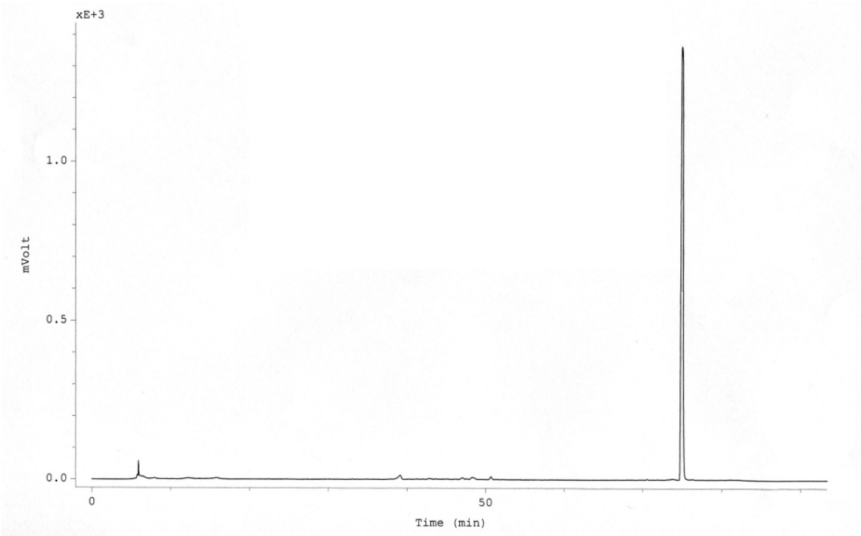

Kaempferol

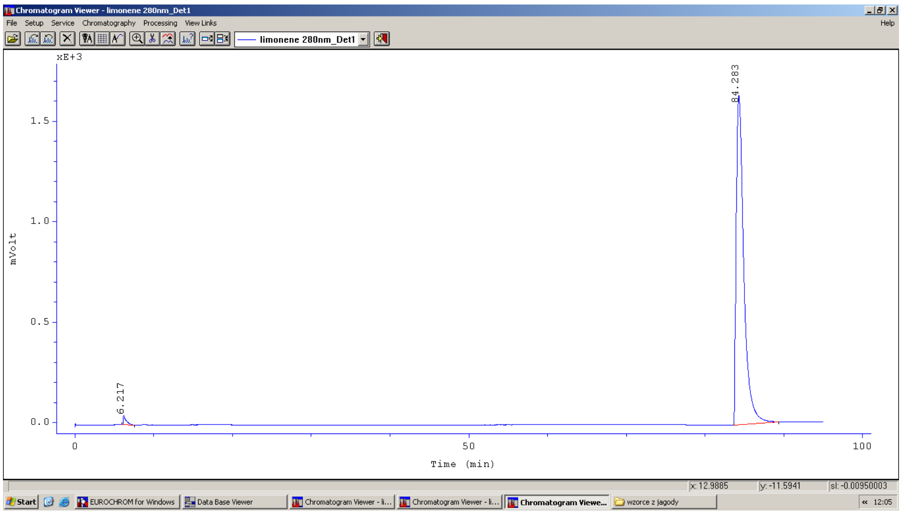

Limonene

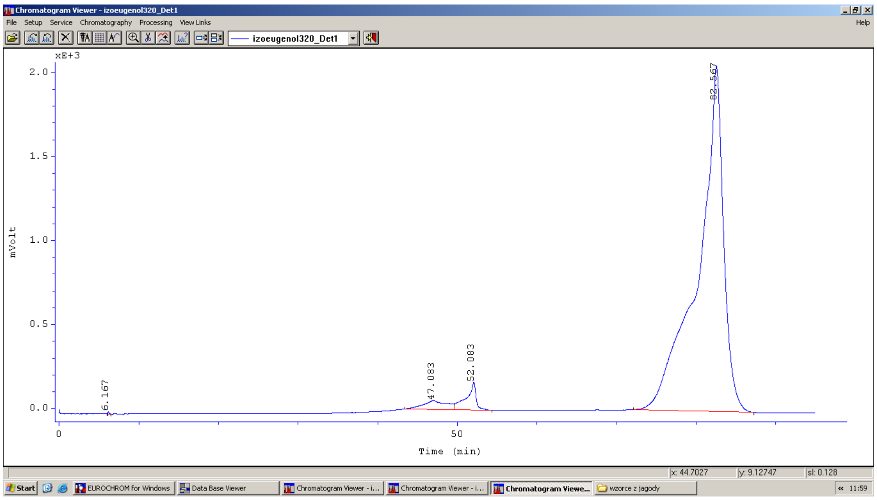

Isoeugenol

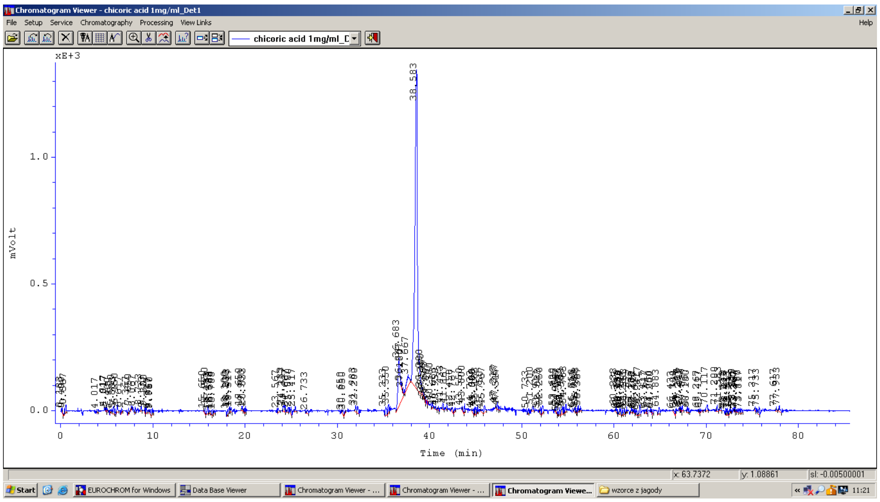

Chicory acid

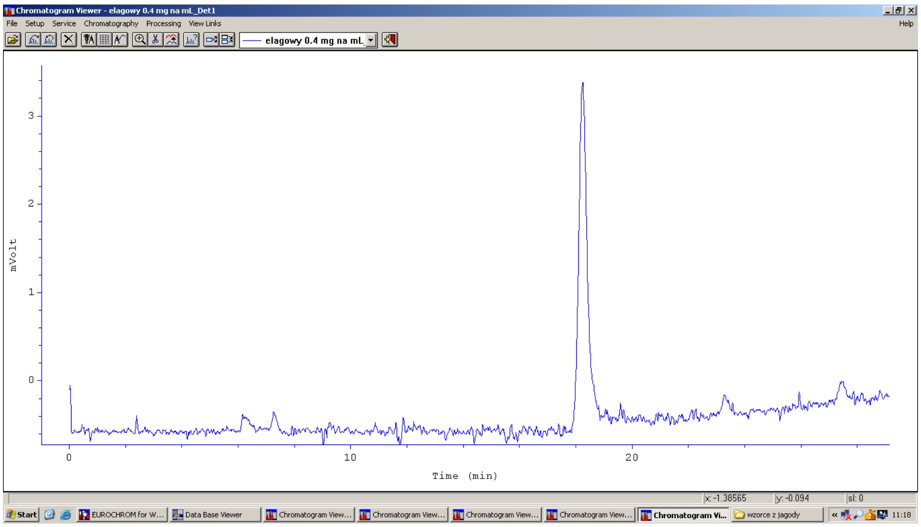

Ellagic acid

Figure S1. Chromatograms of HPLC standards.

Selected chromatograms of ultrafiltrates from fruits (HPLC-C18) are presented below.

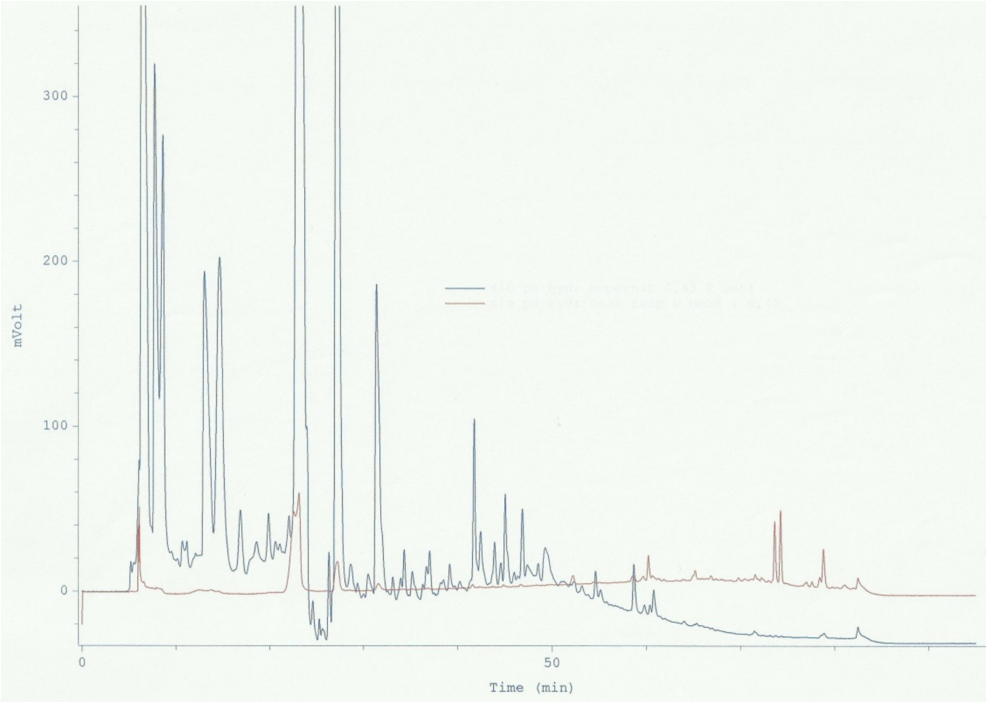

•
Figure S2. Apricot—compounds present in the ultrafiltrate (5 kDa); analytical HPLC-UV at 280 nm.

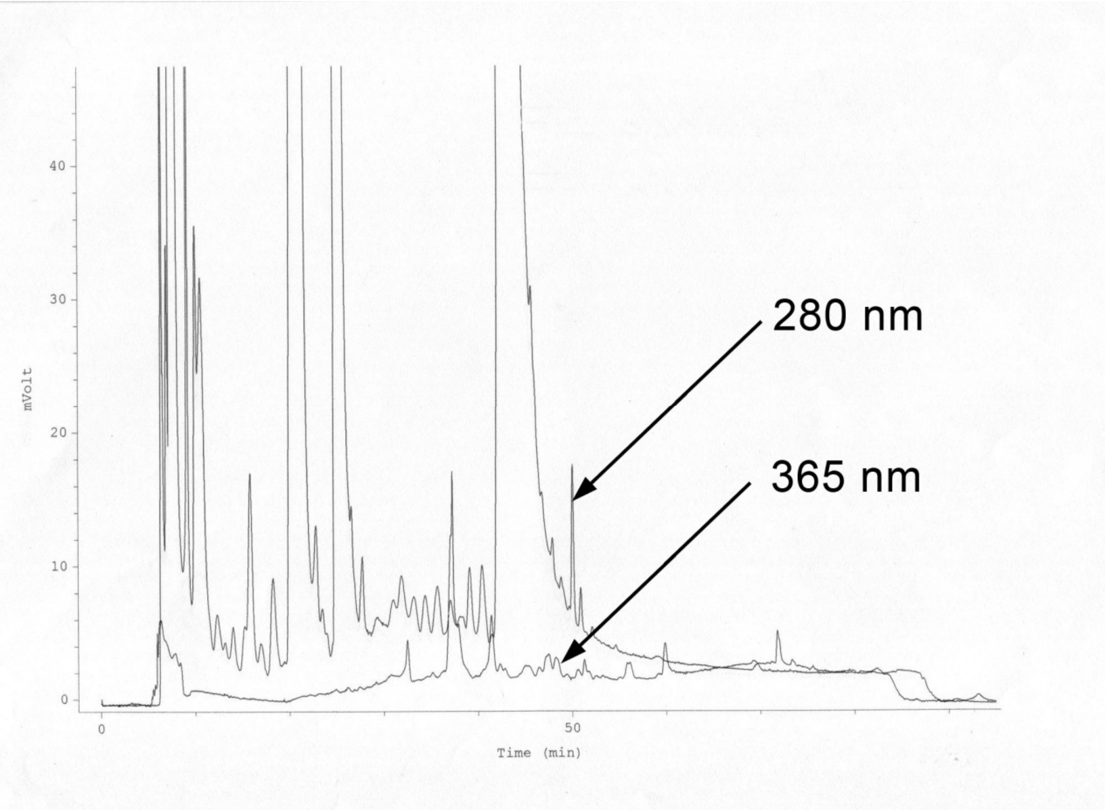

Figure S3. Peach—compounds present in the ultrafiltrate (5 kDa); analytical HPLC-UV at 280 nm.

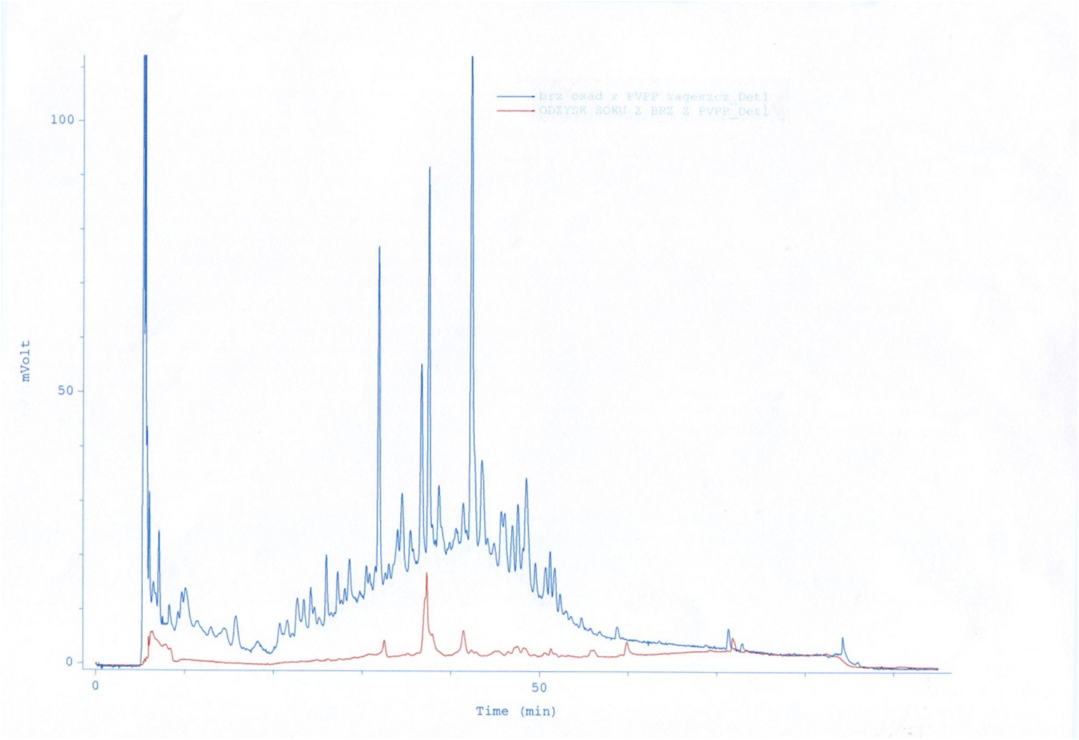

Figure S4. Phenolic compounds from peach separated by polyvinylpolypyrrolidone (PVPP); analytical HPLC, 280 nm.
Blue line, phenolic compounds adsorbed to PVPP; red line, phenolic compounds that remained in solution = not adsorbed to PVPP

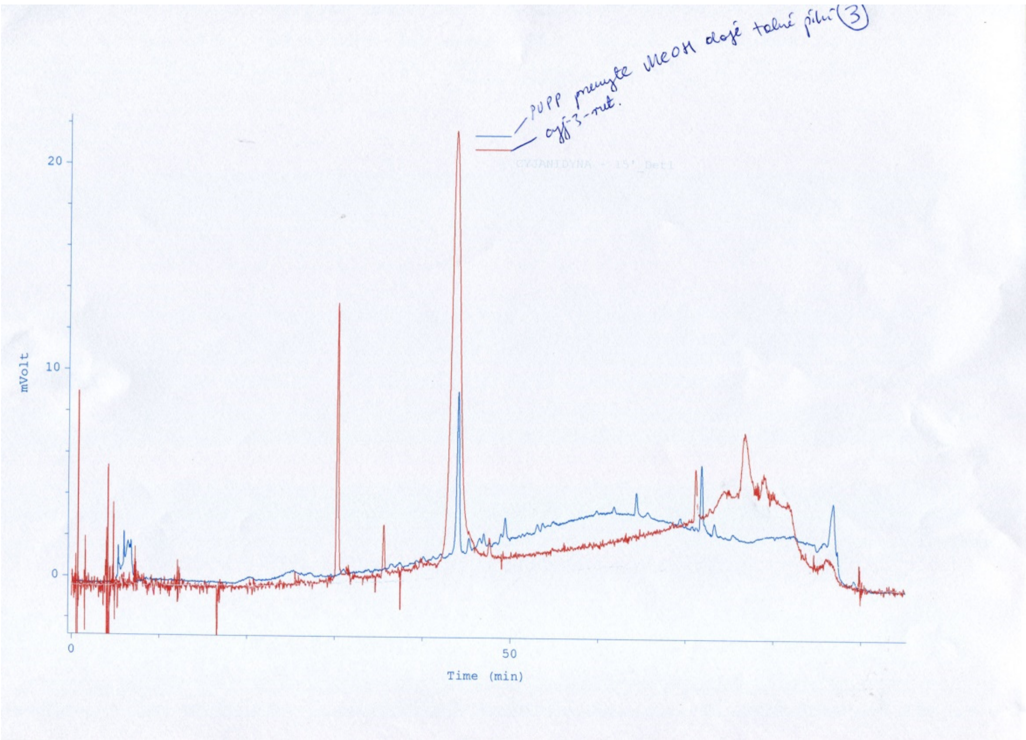

Figure S5. Example of identification of a phenolic compound (cyanidin-3-rutinoside) from peach extract. Cyanidin-3-rutinoside was purified by adsorption on PVPP followed by analytical HPLC chromatography.

Red line, HPLC standard; blue line, chromatogram of compounds isolated from peach ultrafiltrate.

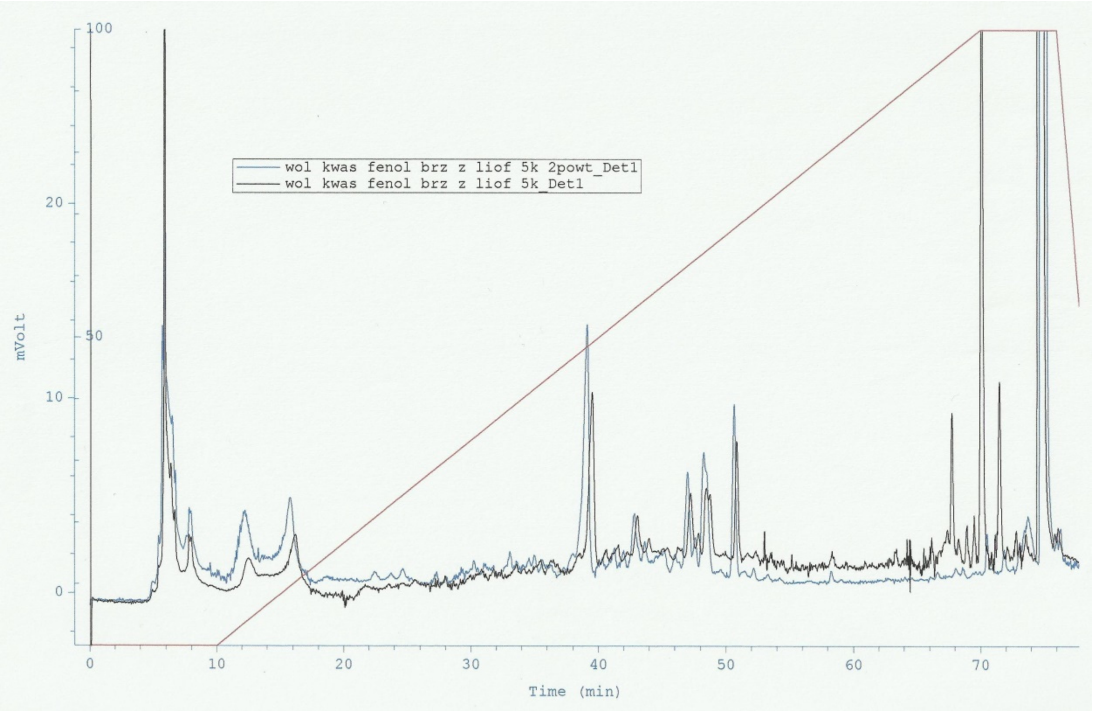

Figure S6. Free phenolic acid present in peach ultrafiltrate (5kDa), 280 nm (two independent extractions).

Figure S7 shows an example of a chromatogram of the final PP.

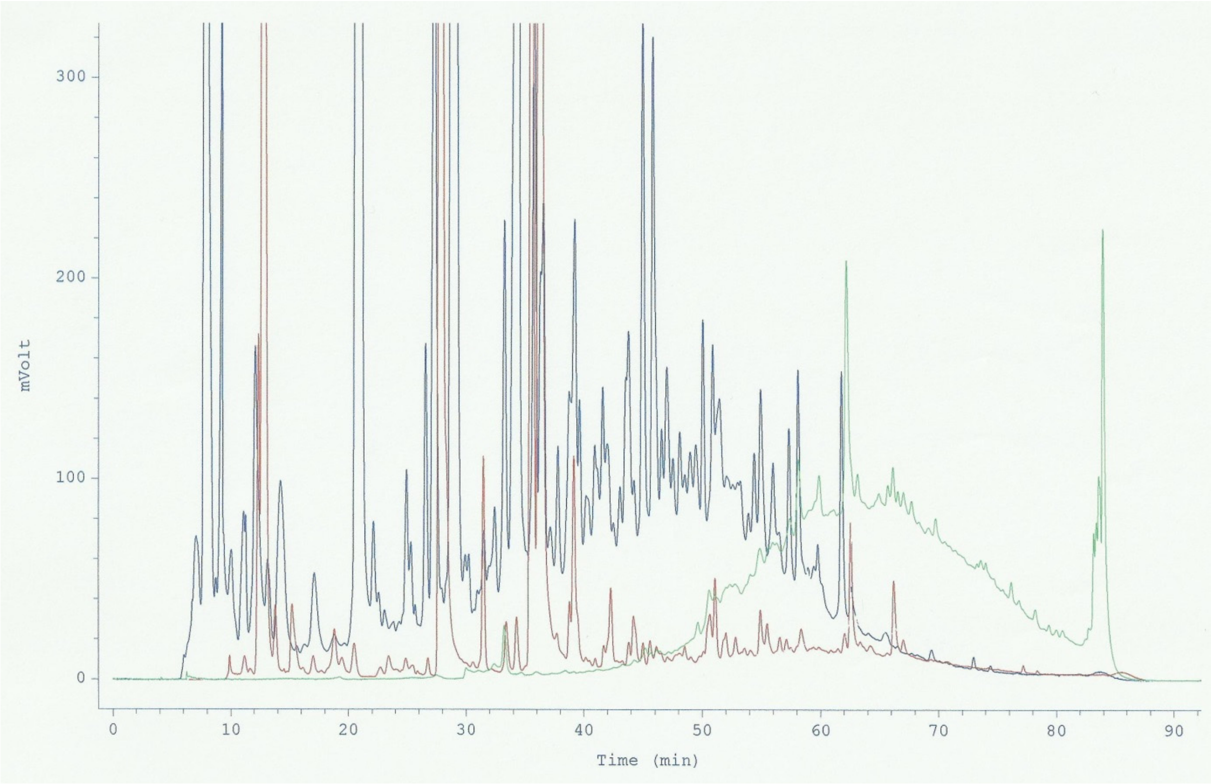

Figure S7. PP chromatogram (analytical HPLC-UV detection at 280 nm (blue), 365 nm (green), and 520 nm (red)).

Qualitative and quantitative analysis of WZ HPLC-HRMS and HRMS/MS

Quantitative analysis allowed determining the concentration of the most quantitatively important compounds in the extract (Figure S8 and Table S2).

<table>
<tr><th>Compound</th><th>MS/MS Spectrum</th></tr>
<tr><td>Chlorogenic acid</td><td>
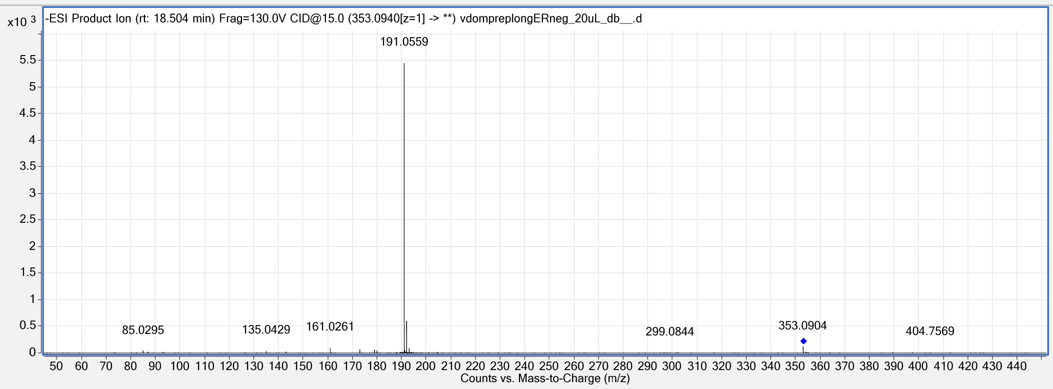

</td></tr>
<tr><td>Protocatechuic acid</td><td>
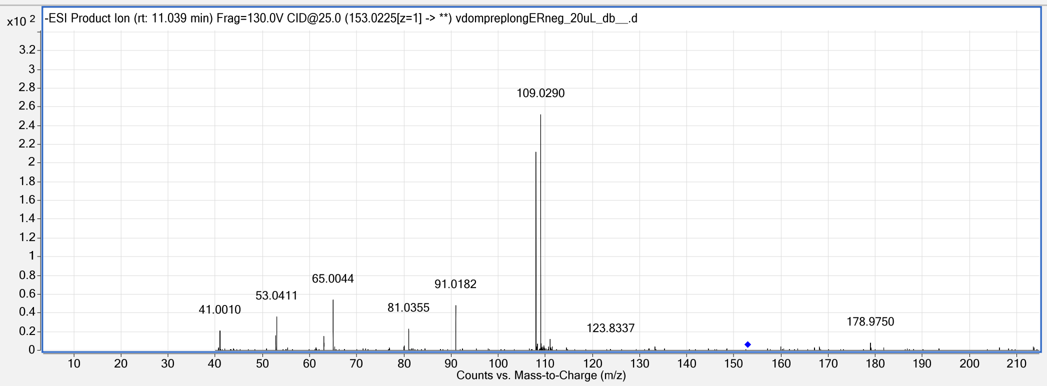

</td></tr>
<tr><td>Coumaric acid</td><td>
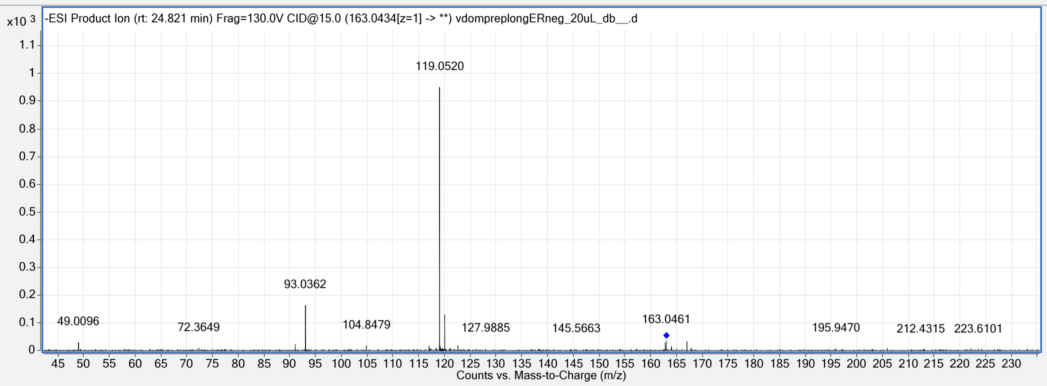

</td></tr>
</table>

Caffeic acid

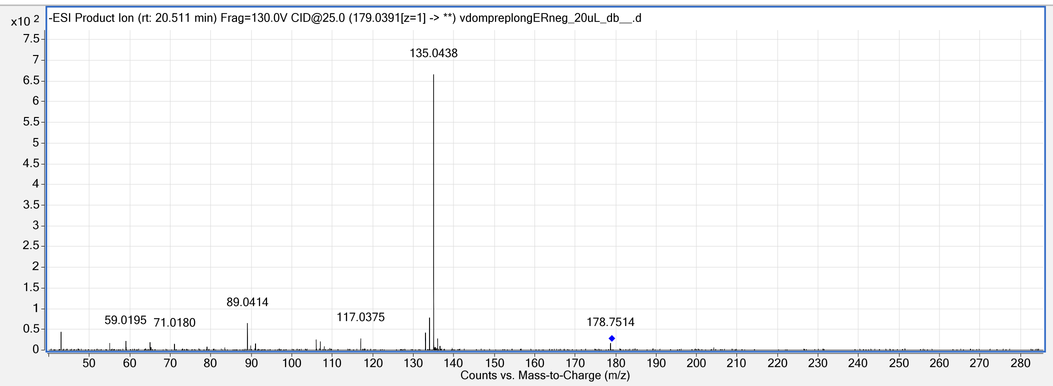

Gentisic acid

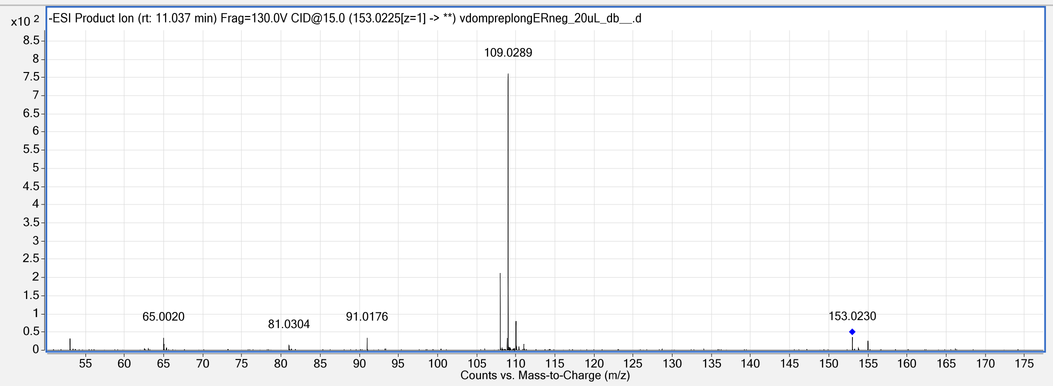

Cyanidin 3-glucoside

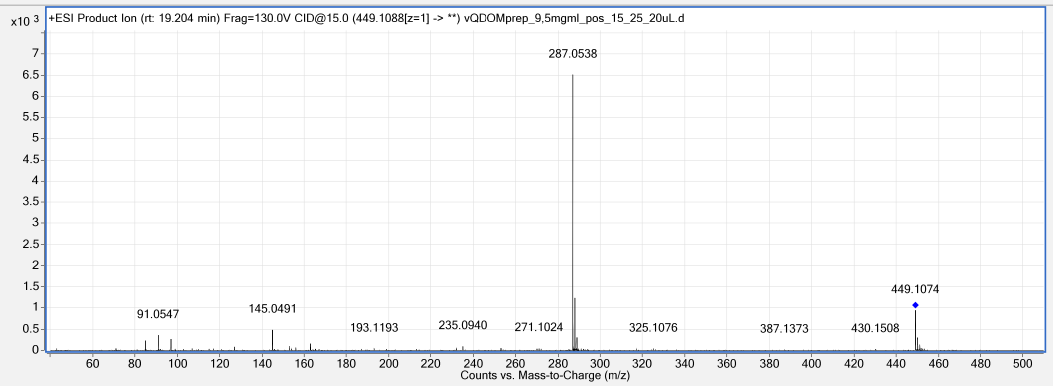

4-OH-Benzoic acid

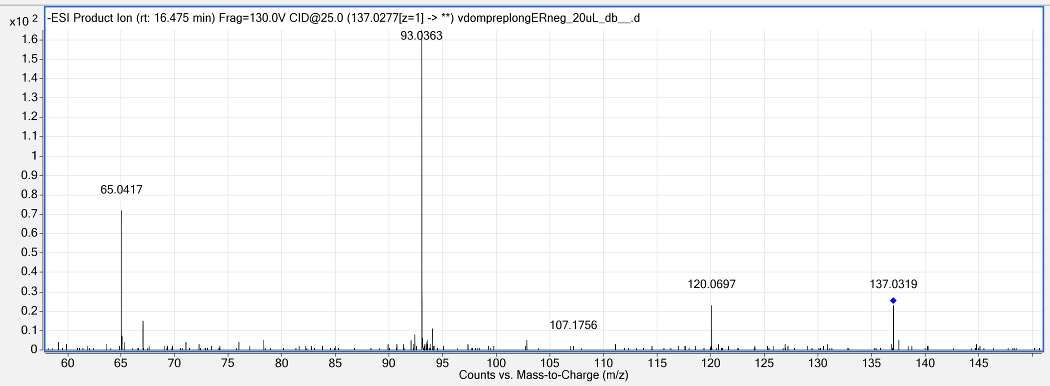

<table>
<tr><td>3-OH-Benzoic acid</td><td>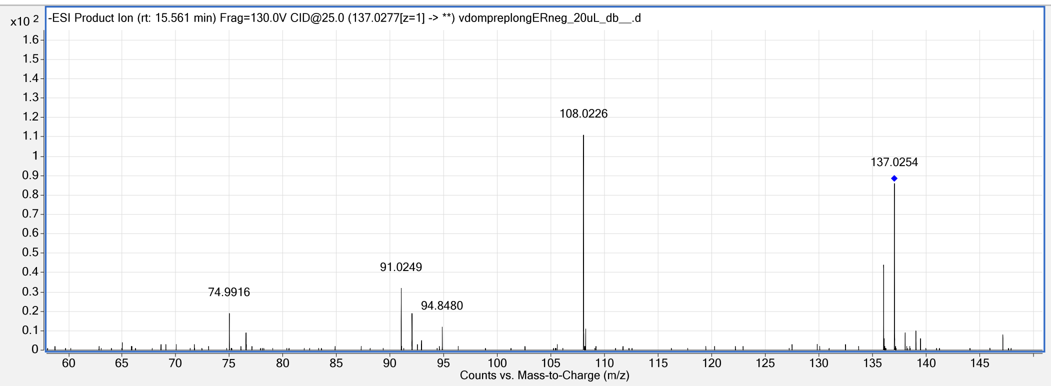
</td></tr>
<tr><td>2-OH-Benzoic acid derivative</td><td>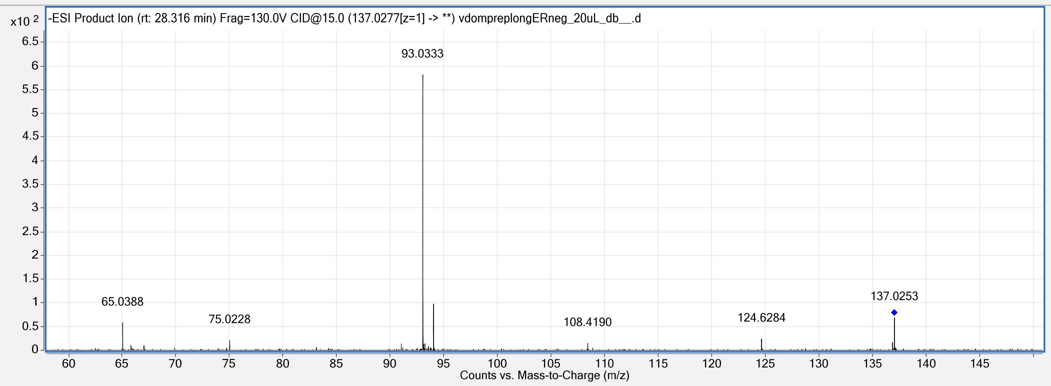
</td></tr>
</table>

Figure S8. Example MS/MS spectra of compounds present in PP.

Table S2. Retention times of authentic HPLC standards and fragmentation parameters of the main polyphenolic compounds detected in PP.

Ion (+/-)	*Retention time (min)*	*Summary formula*	*Experimental m/z*	*Calculated m/z*	*Delta (mmu)*	*RDB*	*MS/MS fragments*	*Proposed compound*
-	4.8	$C_7H_6O_5$	169.0153	169.0142	-6.19	5.0	-	Gallic acid
-	10.8	$C_7H_6O_4$	153.0200	153.0193	-4.34	5.0	109, 91, 65	Gentisic acid
-	11.1	$C_7H_6O_4$	153.0188	153.0193	3.46	5.0	109, 91, 65, 53	Protocatechuic acid
-	14.0	$C_{16}H_{18}O_9$	353.0877	353.0878	0.3	8.0	191, 179, 135	Neochlorogenic acid
-	15.5	$C_7H_6O_3$	137.0245	137.0244	-0.6	5.0	108, 94, 91	3-Hydroxybenzoic acid
-	16.5	$C_7H_6O_3$	137.0245	137.0244	-0.6	5.0	120, 107, 93, 65	4-Hydroxybenzoic acid
-	18.4	$C_{16}H_{18}O_9$	353.0895	353.0878	-4.78	8.0	191, 161, 135	Chlorogenic acid
+	18.9	$C_{27}H_{30}O_{16}$	611.1613	611.1607	-1.05	13.0	565, 433, 287, 227, 116	Kaempferol-3-sophoroside derivative
+	19.2	$C_{21}H_{21}O_{11}$	449.1074	449.1078	0.98	12.0	287, 235, 145	Cyanid 3-glucoside
-	19.4	$C_8H_8O_4$	167.0367	167.0350	-10.2	5.0	-	Vanillic acid
-	20.3	$C_9H_8O_4$	179.0360	179.0350	-5.65	6.0	135, 117, 89	Caffeic acid
-	23.3	$C_{17}H_{20}O_9$	367.1042	367.1035	-2.02	8.0	249, 191, 173, 93	4-O-Feruloquinic acid
-	23.7	$C_{15}H_{18}O_8$	325.0943	325.0956	-4.32	7.0	163, 119, 91, 71	4-coumaryl-glucoside
-	24.5	$C_8H_8O_4$	167.0349	167.0350	0.49	5.0	-	Isovanillic acid
-	24.9	$C_9H_8O_3$	163.0420	163.0401	-11.78	6.0	119, 104, 93	Coumaric acid
+	25.1	$C_{21}H_{21}O_{10}$	433.1147	433.1129	-4.11	12.0	365, 301, 229, 133	Quercetin derivative

-	25.5	$C_{18}H_{16}O_8$	359.0737	359.0772	9.83	11.0	197, 153, 135, 89	Rosmarinic acid
-	26.7	$C_{10}H_{10}O_4$	193.0499	193.0506	3.77	6.0	-	Ferulic acid
+	27.4	$C_{21}H_{24}O_{10}$	437.1427	437.1442	3.49	10.0	-	Phloretin 2′-β-D-glucoside (Phloridzin)
-	28.4	$C_7H_6O_3$	137.0241	137.0244	2.3	5.0	108, 93, 75, 65	2-Hydroxybenzoic acid derivative
-	28.7	$C_9H_8O_2$	147.0439	147.0452	8.46	6.0	-	Cinnamic acid
+	30.7	$C_{15}H_{10}O_7$	303.0508	303.0499	-2.88	11.0	285, 257, 229, 165, 153, 137	Quercetin
+	31.4	$C_{15}H_{11}O_7$	303.0489	303.0499	3.41	11.0	-	Delphinidin
-	31.7	$C_{11}H_{12}O_5$	223.0633	223.0612	2.58	6.0	-	Sinapic acid
+	31.8	$C_{15}H_{10}O_8$	319.0456	319.0448	-2.38	11.0	-	Myricetin
+	35.5	$C_{21}H_{20}O_{12}$	465.1039	465.1028	-2.47	12.0	333, 303, 257, 165, 127	Quercetin 3-glucoside (Isoquercetin)
+	36.9	$C_{27}H_{30}O_{15}$	595.1613	595.1626	7.48	13.0	435, 398, 223, 287, 147	Kaempferol 3-rutinoside
+	38.2	$C_{22}H_{23}O_{12}$	479.1175	479.1190	1.89	12.0	-	Petunidin-glucoside
+	43.4	$C_{30}H_{26}O_{12}$	597.1532	579.1497	-6.05	18.0	-	Procyanidin B2

Table S3. Quantitative analysis of selected compounds present in PP.

Compound	Ion (+/-)	Calibration curve	R^2	Identified compound	% content in the sample	SD
Quercetin	+	y = 53,760,657x + 8,171,143	0.9949	Quercetin derivative<br>Isoquercetin	0.85<br>1.97	0.064<br>0.11
Naringenin	+	y=52,263,581x + 637,745	0.9984	Cya-3-glu	8.18	0.38
				Kaempferol-rut	0.014	0.001
Chlorogenic acid	-	y = 47,887,804x + 240,274	0.9960	Chlorogenic acid	6.35	0.23
				Neochlorogenic acid	4.85	0.31
Protocatechuic acid	-	y = 31,018,319x + 1,007,975	0.9987	Protocatechuic acid	0.53	0.02
4-Coumaric acid	-	y = 25,723,840x - 872,820	0.9977	4-Coumaric acid<br>p- Coumaryl-glu	0.24<br>7.96	0.007<br>0.51
Gentisic acid	-	y = 16,868,073x - 941,479	0.9996	Gentisic acid	1.053	0.048

Table S4. Gelling agents used.

Gelling agents used	Conc. used (%m/m)
Agar (E406, Agnex, Białystok, Poland)	1
Amidated pectin with dextrose	3 + 0.1 Ca^{2+} ions
Carragenean with xanthan gum	0.5 + 0.1 K^{+} ions
Highly methylated pectin	3
Pork gelatin	10
Pork gelatin + cellulose	10 + 2
Pork gelatin + guar gum	10 + 1
Pork gelatin + locust bean gum	10 + 1
Pork gelatin + methylcellulose	10 + 2
Pork gelatin + starch (ViVio, Poland)	10 + 2
Pork gelatin + starch (WarChem, Polska)	10 + 2
Pork gelatin + xanthan gum	10 + 1
Sodium alginate + sodium caseinate	7 (in the ratio of 1:3)

Table S5. Significant disintegration of jellies during in vitro digestion.

**Gelling agents used**	**Overall estimated significant disintegration**
Agar (E406, Agnex, Białystok, Poland)	1
Amidated pectin with dextrose	„small intestine”, 40 min
Carragenean with xanthan gum	„stomach”, 10 min
Highly methylated pectin	„small intestine”, 20 min
Pork gelatin	„small intestine”, 80 min
Pork gelatin + cellulose	„small intestine”, start
Pork gelatin + guar gum	„small intestine”, 20 min
Pork gelatin + locust bean gum	„small intestine”, 40 min
Pork gelatin + methylcellulose	„small intestine”, start
Pork gelatin + starch (ViVio, Poland)	„small intestine”, start
Pork gelatin + starch (WarChem, Polska)	„small intestine”, start
Pork gelatin + xanthan gum	„small intestine”, 40 min
Sodium alginate + sodium caseinate	„small intestine”, start

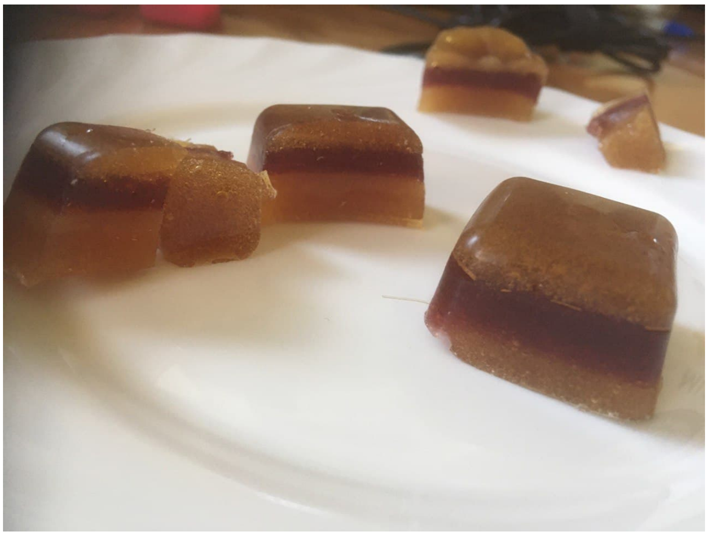

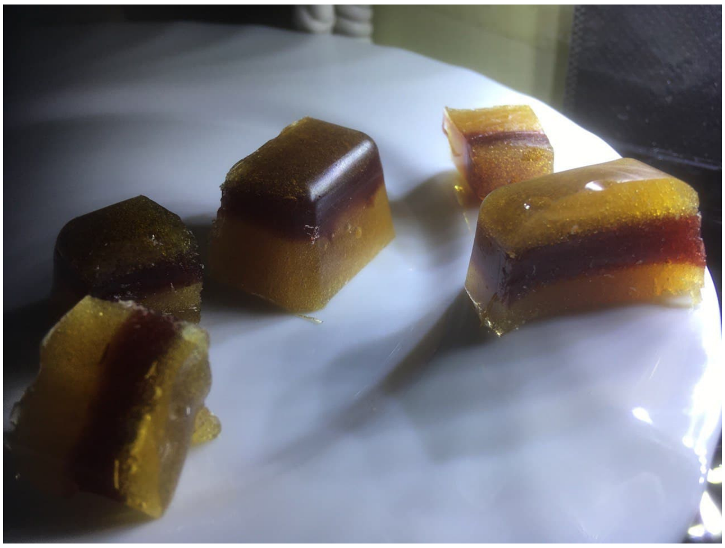

Figure S9. Prototype jellies produced using gelatin.

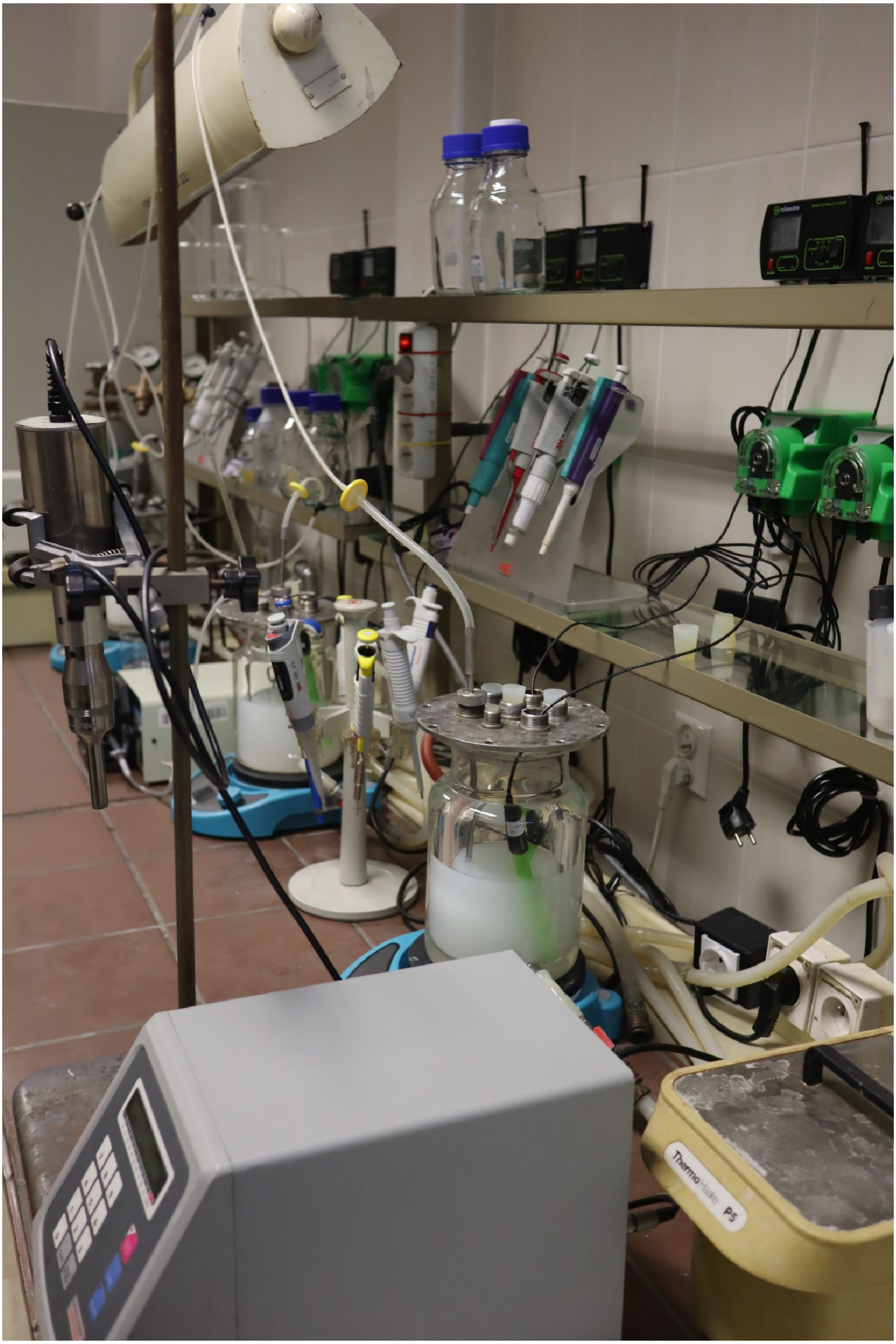
ThermoHaake P5

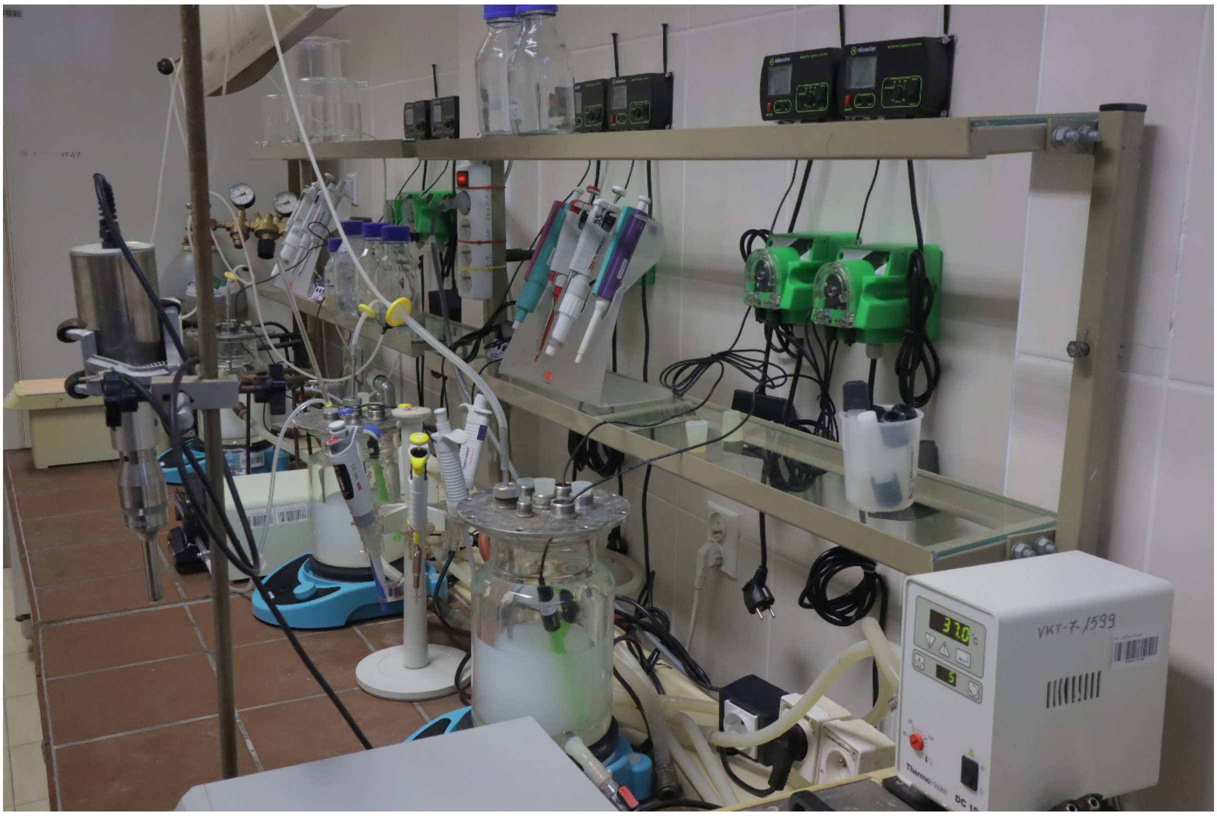

Figure S10. In vitro digestion model used in this study.

Table S6. Dry mass of samples during in vitro digestion.

Digestion stage	%		
	Digestive fluids)	Crude PP	PP in jellies
Stomach start	4.0 ± 0.1	7.6 ± 0.1	1.3 ± 0.1
Stomach 10 min	4.2 ± 0.2	7.7 ± 0.3	5.0 ± 0.1
Stomach 20 min	4.0 ± 0.1	7.3 ± 0.1	8.6 ± 0.2
Small intestine start	4.1± 0.2	6.4 ± 0.1	9.8 ± 0.3
Small intestine 20 min	4.3 ± 0.2	6.6 ± 0.3	11.9 ± 0.0
Small intestine 40 min	4.2 ± 0.1	6.7 ± 0.1	14.5 ± 0.1
Small intestine 60 min	4.9 ± 0.2	7.0 ± 0.2	14.5 ± 0.2
Small intestine 80 min	4.3 ± 0.0	7.5 ± 0.1	14.5 ± 0.2
Small intestine 100 min	4.0 ± 0.2	7.1 ± 0.1	14.9 ± 0.1

n=4 ± SD
